# Tumor growth monitoring in breast cancer xenografts: A good technique for a strong ethic

**DOI:** 10.1371/journal.pone.0274886

**Published:** 2022-09-30

**Authors:** Anne Rodallec, Cristina Vaghi, Joseph Ciccolini, Raphaelle Fanciullino, Sebastien Benzekry

**Affiliations:** 1 COMPO, CRCM, INRIA Sophia Antipolis, INSERM UMR1068, CNRS UMR7258, AMU U105, IPC, Marseille, France; 2 SMARTc, CRCM, INSERM UMR1068, CNRS UMR7258, AMU U105, IPC, Marseille, France; 3 MONC, Inria Bordeaux Sud-Ouest, Talence, France; Dartmouth College Geisel School of Medicine, UNITED STATES

## Abstract

**Purpose:**

Although recent regulations improved conditions of laboratory animals, their use remains essential in cancer research to determine treatment efficacy. In most cases, such experiments are performed on xenografted animals for which tumor volume is mostly estimated from caliper measurements. However, many formulas have been employed for this estimation and no standardization is available yet.

**Methods:**

Using previous animal studies, we compared all formulas used by the scientific community in 2019. Data were collected from 93 mice orthotopically xenografted with human breast cancer cells. All formulas were evaluated and ranked based on correlation and lower mean relative error. They were then used in a Gompertz quantitative model of tumor growth.

**Results:**

Seven formulas for tumor volume estimation were identified and a statistically significant difference was observed among them (ANOVA test, p < 2.10^−16^), with the ellipsoid formula (1/6 π × L × W × (L + W)/2) being the most accurate (mean relative error = 0.272 ± 0.201). This was confirmed by the mathematical modeling analysis where this formula resulted in the smallest estimated residual variability. Interestingly, such result was no longer valid for tumors over 1968 ± 425 mg, for which a cubic formula (L x W x H) should be preferred.

**Main findings:**

When considering that tumor volume remains under 1500mm^3^, to limit animal stress, improve tumor growth monitoring and go toward mathematic models, the following formula 1/6 π × L × W x (L + W)/2 should be preferred.

## Introduction

The year 2020 was marked by a revised version of the ARRIVE (Animal Research: Reporting In Vivo Experiments) guidelines 2.0, elaborated to pursue our efforts in improving the reproducibility of biomedical research while respecting animal welfare [1]. This work constitutes an instruction update of the Essential 10 (i.e., study design, sample size, inclusion and exclusion criteria, randomization, blinding, outcome measures, statistical methods, experimental animals, experimental procedures, and results) that are necessary to every publication for best practice in animal research.

Indeed, although it is restricted to specific fields with more regulations and number of subjects has been reduced [2], animal research remains a fundamental step in biomedical research including cancer that constitutes the largest portion in translational and applied research [3].

Among all studied cancers, breast cancer remains the most common and deadliest for women worldwide and animal models are crucial for its treatment perspectives [4]. Many experimental models have been developed on non-mammal and mammal species, such as spontaneous, induced, transplanted and genetically engineered models [4], which complexification increases efficacy prediction in the clinic. However, such developments can be expensive, time consuming and clinically irrelevant [5]. Thus, xenograft orthotopic implantations, the most popular animal model for testing new therapies, appear as an interesting compromise since it is neither time consuming nor cost effective and it is representative of the clinical tumor’s environment [4,5].

**Fig 1:**
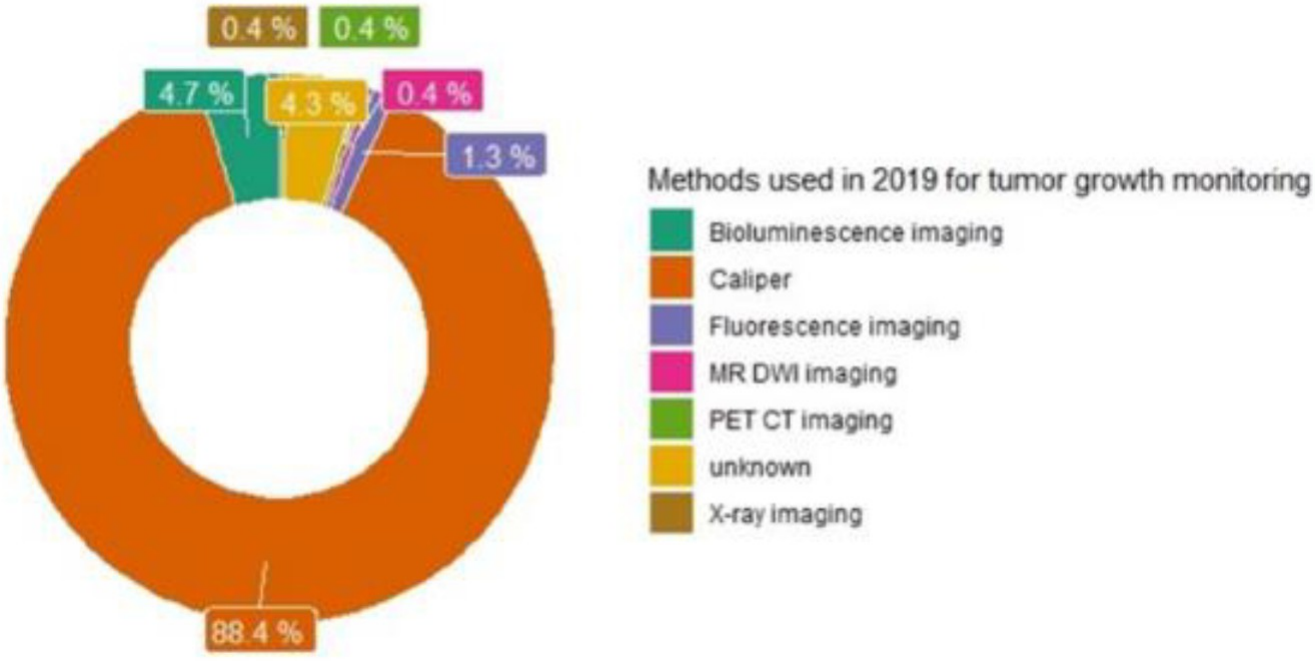
Representation of all techniques used in 2019 for tumor growth monitoring on human breast cancer xenografts bearing mice (n = 233). The *bibliographic search was conducted in the PubMed database from January 1*^*st*^, *2019 to December 31*^*st*^, *2019*, *using the followings terms in Text Word*: X*enograft*, *breast cancer*, *tumor growth AND mice*. *MR DWI = *Diffusion-weighted* magnetic resonance; PET CT = positron emission tomography computing tomography; Unknown = information not accessible.

Based on these facts, and on the largest use of mice in oncology [3], we evaluated all existing techniques for tumor growth volume monitoring, used on human breast cancer xenografts bearing mice in 2019 and presented their varieties in Fig 1. Although, caliper measurement is the oldest technique and cannot evaluate metastasis spreading, because of cost, non-invasive and ease of use [6], it was found to remain the most popular one (i.e., 88.4%).

Caliper measurement is a rapid technique that assesses tumor growth and disease progression by collecting dimensions of the tumor (i.e., L = length, W = width, H = height or depth) and using specific formulas to define tumor volume. Such metric is indeed fundamental, not only for its purpose in quantifying response to therapeutic regimen but also because it can define humane endpoint for determining when animal welfare is at risk and animal should be sacrificed. However, when investigating this approach more closely, many formulas are being used to estimate tumor volume and no standardization is available yet. Thus, in line with the ARRIVE guidelines 2.0 [1] and in the framework of helping investigator’s methodological rigor, we offer here a statistical analysis of all formulas used in 2019 and an attempt towards standardization. No additional animal study was performed for this work, and all presented tumor growth data come from previous experiments that collected tumor dimensions on human breast cancer xenografts bearing mice using manual caliper and were compared to the most accurate tumor size metric: tumor mass.

Since quantitative modeling allows to reduce drastically animal experiments and suffering, we also tested the impact of tumor volume estimation on the population analysis of the Gompertz model. Tumor growth kinetics has been extensively studied by means of mathematical equations which describe the dynamic of cancer growth [7]. However, mathematical models must be validated against reliable data to provide good insights on the mechanism of tumor growth and to improve future predictions. The Gompertz model has been extensively applied in tumor growth kinetics and has shown good descriptive and predictive performances on several cancer types in different species [8–10].

## Materials and methods

### Identification of all existing formulas for tumor volume estimation

A bibliographic search was conducted in the PubMed database from January 1^st^, 2019 to December 31^st^, 2019, using the followings terms in Text Word: xenograft, breast cancer, tumor growth AND mice. Only articles displaying caliper measurements used for tumor volume monitoring were included for the rest of the study.

### Animal ethic

No additional animal was used for this study and all data come from previous works, submitted, and validated by the Animal Ethic Committee of Aix Marseille Université (CE14). The protocols were registered as #2017031717108767 and #20181213155790720 at the French ministry of research and followed the Directive 2010/63/EU for the care of used mice. Mice were kept 5 per cage with specific cotton and housing with unlimited access to water and food. Metastasis related pain was prevented using 80 mg/kg/day paracetamol supplemented water. Mice were monitored daily to look for humane endpoints such as weight loss, loss of mobility, tumor volume > 2000 mm^3^ etc. When humane endpoint was reached mice were sacrificed after anaesthesia (i.e., 3% sevoflurane) by cervical dislocation.

### Tumor growth monitoring

Depending on availability, data from up to 93 female Swiss nude *nu/nu* mice (Charles River, France) were collected. As previously published mice were orthotopically xenografted (mammary fat pad) with 80000 human breast cancer cells, (i.e., MDA MB 231) using 60% matrigel matrix [11]. Tumor dimensions (i.e., length, width, and measurable height) were measured twice a week using manual caliper. Once humane endpoint reached and animal sacrificed, we performed tumor exeresis, weighted and measured dimensions (i.e., length, width, and height) of the tumor. To evaluate accuracy of caliper measurements in living animals, statistical analysis between dimension measurement performed before and after exeresis was conducted (i.e., correlation and mean relative error).

### Tumor volume formula analysis

Statistical, linear regression and correlation analysis was performed on R-4.0.3. using *lm()*, *abline()*, *deviance()* and *summary()* functions. *Lm()* function was used to carry out regression and fit linear models, *abline()* function was used as a control to add the line y = x, *deviance()* function was used to return the deviance between this control and the linear model and *summary()* function was used to extract regression results such as r^2^, *p* or the slope of regression line (i.e., *a*). If p is found < 0.05, the two variables are correlated. The greater r^2^ is, the stronger the correlation is.

All formulas identified from our bibliographic search were tested on our collected data (S2 Table) and compared to tumor mass using the following equivalences: 1gr = 10^9^ cells; 10^6^ cells = 1mm^3^ [12]. Ranking system was based on lower mean relative error (MRE) and r^2^.

### Impact of tumor size on tumor volume estimation

To evaluate impact of tumor size on tumor volume estimation, we divided our data into two populations: the smaller 50% and the larger 50% tumor mass (i.e., small tumor size population and large tumor size population) and repeated identical analysis than described above.

### Impact of tumor volume estimation on the population analysis of the Gompertz model

Denoting *V*(*t*) the tumor volume at time *t*, the Gompertz model main assumption is that cancer cells initially grow exponentially with a specific growth rate (i.e. 1/*V*⋅*dV*/*dt*) *α*, which decreases exponentially in time with rate *β*. Writing this as an autonomous ordinary differential equation, the model reads

$$dV/dt=\left(\alpha -\;\beta \;V/V\_inj\right)V$$

where *V_inj* is the experiment-specific constant of the volume of injected cells at time *t* = 0. In our case, the number of injected cells was 80000 and thus we assumed *V_inj* = 0.08 mm^3^. The initial condition of this differential equation will be defined below.

We fitted the Gompertz model against the data obtained with the tumor volume formulas. We employed the nonlinear mixed effects modeling approach to analyze the entire population [13]. This statistical framework allowed us to analyse jointly the global dynamic of the population and the inter-individual variability. We denoted by *y_j^i* the volume observation of an animal *i* at time *t*_*j*^*i*, where *i* = 1, …, *N* (*N* is the total number of animals) and *j* = 1, …, *n*^*i* (*n*^*i* is the number of measurements of animal *i*). The following observation model was assumed:

y_j^i=Vt_j^i;α^i,β^i+e_j^i,i=1,…,N,j=1,…,n^i,

where *V*(*t*_*j*^*i*; *α*^*i*, *β*^*i*) is the structural model (i.e., the evaluation of the Gompertz model at time *t*_*j*^*i* and using the individual parameters *α*^*i*, *β*^*i*), and *e*_*j*^*i* is the residual error model.

The individual parameters *α*^*i* and *β*^*i* depend on fixed effects (*α*_*pop* and *β*_*pop*, respectively), which are fixed within the population, and on random effects (*η*_*α*^*i* and *η*_*β*^*i*, respectively). Random effects were assumed normally distributed with mean zero and covariance matrix Ω:

α^i=α_pop⋅e^η_α,β^i=β_pop⋅e^η_β,η_α,η_β~N0,Ω.

We set as initial condition *V*_*I*^*i* the first observation *y*_1^*i* at time *t*_1^*i* of and individual *i*. To account for uncertainty of the measurement, we assumed 15% of experimental error:

V_I^i=V_I^it_1^i,logV_I^i~Nlogy_1^i,0.15.

The residual error model was assumed to be proportional:

e_j^i=σ⋅ε_j^i⋅Vt_j^i;α^i,β^iε_j^i~N0,1,

where *σ* is the error model parameter. The residual error model represents the unexplained variability of the observation model. It describes variations that arise from several sources (e.g., measurement error, model misspecification). The minimization of the unexplained variability allows to have a better description of the global dynamic and the inter-individual variability with the structural model.

To compare the different volume formulas, we looked at the error model parameter *σ*. In fact, the smaller the value of *σ*, the smaller the unexplained variability of the observations. Furthermore, we analyzed the goodness-of-fit of the Gompertz model using diagnostic plots and assessing the parameter identifiability. All the nonlinear mixed effects modeling analysis was performed using MonolixSuite R2019 [14]. To perform such analysis, a minimum of three longitudinal tumor volume measurements was required. Thus, twelve mice only were included for this study (S2 Table and S1 Fig).

### Statistical analysis

Data were represented as mean ± standard deviation. Relative error (RE) and mean relative error (MRE) were defined as:

$$RE=\left(yi-xi\right)/xi\;\;\;\;\;\;\;\;\;\;\;\;\;\;\;\;MRE=\;\;\left(\overset{n}{\underset{i=1}{\sum }}\left|\left(yi-xi\right)/xi\right|\right)/n$$

Absolute error (AE) and mean absolute error (MAE) were defined as:

$$AE=yi-xi\;\;\;\;\;\;\;\;\;\;\;\;\;\;\;MAE=\;\;\left(\overset{n}{\underset{i=1}{\sum }}\left|yi-xi\right|\right)/n$$

where *yi* is the prediction and *xi* is the real value.

Statistical analyses were performed using R-4.0.3. According to data distribution and sample size, differences between groups were analyzed by Student’s *t*-test or one-way analysis of variance (ANOVA) completed with Tukey Multiple Comparison test when necessary.

## Results

### Identification of all existing formulas for tumor volume estimation

Among the 245 articles that matched our bibliographic research, 12 had to be excluded because animal species did not match (i.e., zebra fish and not mice) or tumor growth was not evaluated (e.g., comparison of tumor weight after exeresis only). Thus, 233 articles were considered, and among them 206 used caliper measurements for tumor growth monitoring (Supplementary data: S1 Table). To limit number of formulas to test, commonly used coefficients 0.4, 0.5 and 0.52 were gathered with real coefficient π/6. Among all selected articles, 11 formulas were identified and 20.4% of the articles did not mention the formula they used, which we summarized in Table 1. By-products of the ellipsoid formula were found to be the most popular (i.e., Formula 4, Formula 5 and Formula 1 with 52.4%, 16.5% and 5.8% frequencies, respectively) and 4 other formulas were described only once or twice. The following formulas *1/2 (L* × *W)*^*2*^, *1/2 L* × *W*^*2*^ × *H*, *π* × *L/2* × *W/2 and 1/2* × *L* ×*W* were gathered as “other” in Table 1 (i.e., 1.9%) because they did not describe a 3-dimensional representation (i.e., a volume in mm^3^), therefore they were not considered for the rest of the study.

**Table 1:** Summarize of the seven formulas used in 2019 for tumor volume monitoring on human breast cancer xenograft bearing mice and their frequency of use (n = 206).

Identification	Formula	Frequency (%)
Formula 1	1/6 π × L x W x H	5.8
Formula 2	L x W x H	0.5
Formula 3	1/6 π x L x W x (L + W) /2	0.5
Formula 4	1/6 π × L x W^2^	52.4
Formula 5	1/6 π × L x W^2^ *with (W < L)*	16.5
Formula 6	3/4 π x L x W^2^	1
Formula 7	L x W^2^	1
Other	-	1.9
MD	-	20.4
**Total**		**100**

Other = formulas did not describe a 3D representation; MD = Missing data.

### Tumor growth monitoring

All data used for further analysis are summarized in supplementary data (S2 Table). Collected mean tumor length was 12.8 ± 2.9 mm, ranged from 6.44 to 19.23 mm. Collected mean tumor width was 11.6 ± 2.6 mm from 6.09 to 18.34 mm. Collected mean tumor height was 8.2 ± 2.1 mm from 4.5 to 16.65 mm. Collected mean tumor mass after exeresis was 1263.7 ± 812.5 mg from 155 to 2976 mg. Collected mean tumor length after exeresis was 17.6 ± 2.1 mm from 12.1 to 21.4 mm. Collected mean tumor width after exeresis was 16.4 ± 1.8 mm from 11.1 to 19.8 mm. Collected mean tumor height after exeresis was 9.1 ± 2.1 mm from 2.3 to 12.6 mm. Length, width and height measured in living animal were all correlated to their measurements after exeresis (i.e., p = 0.012, p = 0.002 and p = 0.005, respectively). Although not statistically significant (one-way ANOVA, p = 0.42), mean relative error was found to be the greatest for height dimension (i.e., 0.214 ± 0.4828). MRE was of 0.121 ± 0.077 and 0.144 ± 0.0862 for length and width, respectively.

### Tumor volume formula analysis

**Fig 2:**
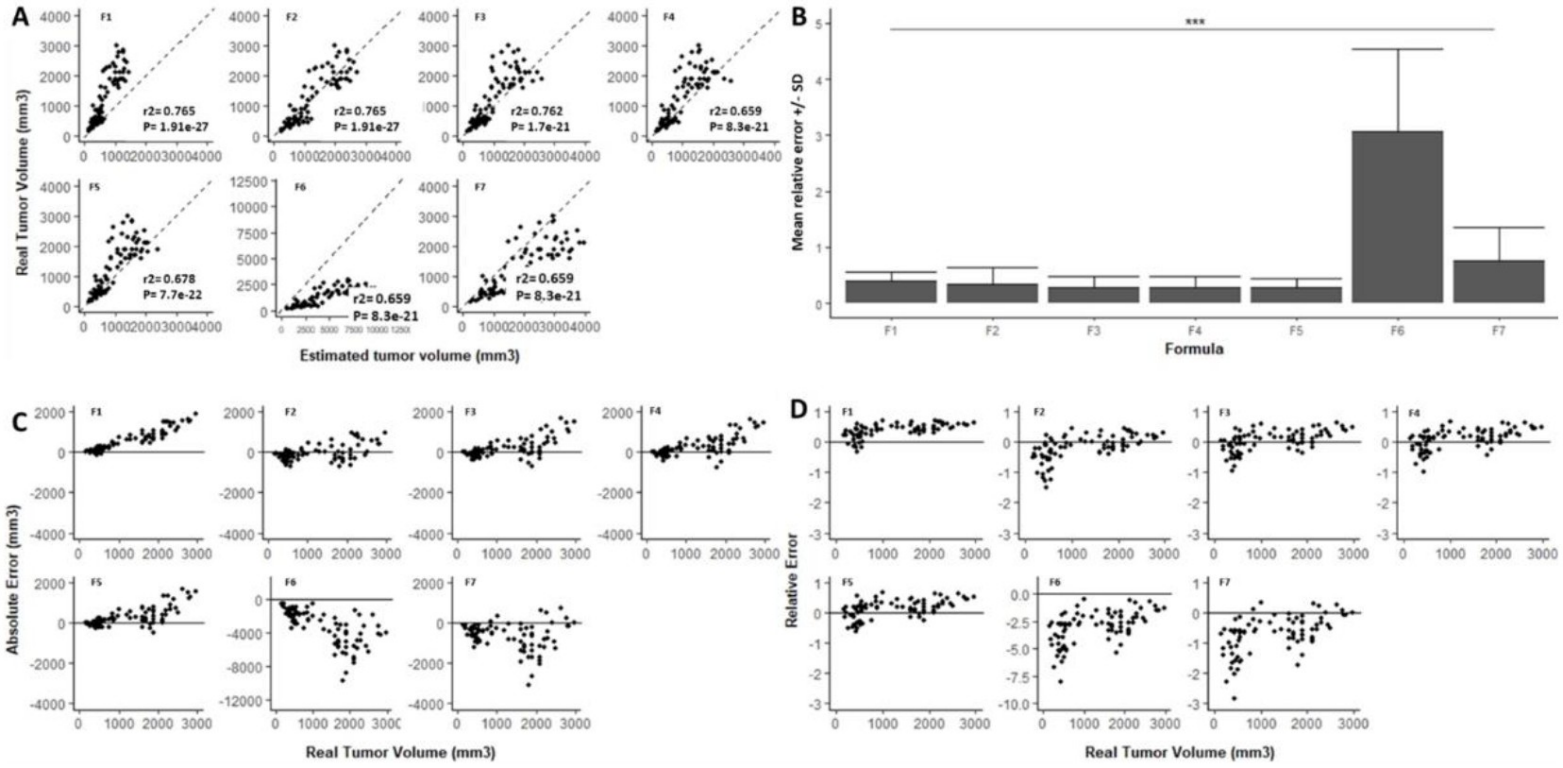
**(A) Correlation graphics between real tumor volume (i.e., tumor mass) and estimated tumor volume with Formula 1 (F1), Formula 2 (F2) Formula 3 (F3), Formula 4 (F4), Formula 5 (F5), Formula 6 (F6) or Formula 7 (F7)** (n = 83). Dotted line is a representation of y = x and the black dots are our observed data (n = 83). **(B) Bar plot of Mean relative error ± SD for all tested volume estimation formulas: F1, F2, F3, F4, F5, F6 and F7 (C) Absolute error sorted by real tumor volume for F1, F2, F3, F4, F5, F6 and F7** (n = 83) **(D) Relative error sorted by real tumor volume for F1, F2, F3, F4, F5, F6 and F7** (n = 83). ***: p = 2.10–16 SD = standard deviation.

Correlation between real tumor volume (i.e., tumor mass) and all seven formulas was confirmed (i.e., p< 0.05) and statistically significant (Fig 2A). The correlation coefficient, r^2^ was the strongest for Formula 2 and Formula 1 (i.e., 0.765). As represented on Fig 2B, MRE were found significantly different among formulas (one-way ANOVA, p = 2.10^−16^) with highest ones for Formula 6, Formula 7, Formula 1 and Formula 2 (i.e., 3.070 ± 1.478, 0.761 ± 0.585, 0.395 ± 0.168 and 0.335 ± 0.313, respectively). Formula 3, Formula 5 and Formula 4 were found to be the most accurate with lowest mean relative errors of 0.272 ± 0.201, 0.275 ± 0.175 and 0.282 ± 0.192, respectively, with no statistically significant difference among them (one-way ANOVA, p = 0.95).

Distribution of AE depending on tumor size was also observed (Fig 2C) and visual analysis showed that for all formulas but Formula 6 and Formula 7, tumor volume were mostly underestimated (i.e., majority of dots are found above the line y = 0) with Formula 2 and Formula 3 presenting the lowest MAE (i.e., 314 ± 240 mm^3^ and 344 ± 375 mm^3^, respectively). When looking at distribution of RE depending on tumor size (Fig 2D), visual analysis showed that Formula 2 lacked of accuracy for small tumor size population, whereas Formula 3 lacked of accuracy for large tumor size population (i.e., dots are further to the line y = 0).

### Impact of tumor size on tumor volume estimation

**Fig 3:**
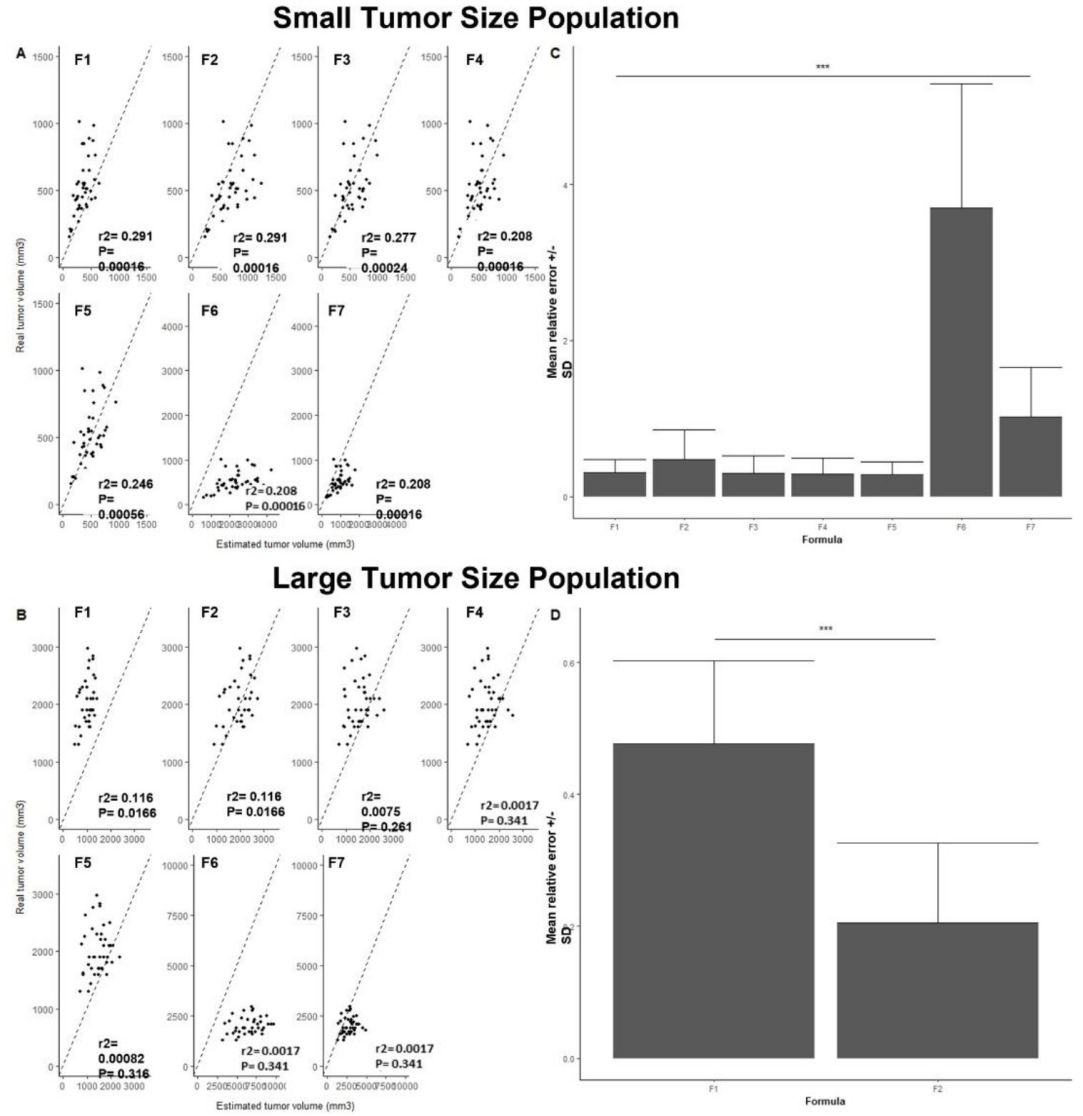
Correlation graphics between real tumor volume (i.e., tumor mass) and estimated tumor volume with F1, F2, F3, F4, F5, F6 or F7 for the small tumor size population (A) and for the large tumor size population (B). Dotted line is a representation of y = x and the black dots are our observed data (n = 41). *Bar plot of Mean relative error ± SD for all tested volume estimation formulas*: *F1*, *F2*, *F3*, *F4*, *F5*, *F6 or F7 for the small tumor size population (C) and for the large tumor size population (D) (n = 41)*. *SD = standard deviation *** = p < 0.*001*.

Mean tumor mass for the small tumor size population and the large tumor size population were 520.54 ± 212.52 mg, ranged from 155 to 1011 mg and 2008.4 ± 416.77 mg, ranged from 1304 to 2976 mm, respectively. While all formulas were correlated with real volume for the small tumor size population (Fig 3A), only Formula 1 and Formula 2 were correlated for the large tumor size population (Fig 3B) with mean relative errors of 0.477 ± 0.126 and 0.205 ± 0.121, respectively (Fig 3D, Student’s *t*-test, p = 5.10^−15^). For the small tumor size population, mean relative errors were found significantly different among tested formulas (one-way ANOVA, p = 2.10^−16^) with highest ones for Formula 6, Formula 7, Formula 2 and Formula 1 (Fig 3C, i.e., 3.070 ± 1.59, 1.02 ± 0.640, 0.472 ± 0.385 and 0.309 ± 0.166, respectively). Still for this population, Formula 5, Formula 4 and Formula 3 were found to be the most accurate with lowest mean relative errors of 0.275 ± 0.167, 0.290 ± 0.203 and 0.295 ± 0.226, respectively (Fig 3C, One-way ANOVA, p = 0.91). These results were found to be true until mean tumor mass of the small tumor size population remains under 1968 ± 425 mg.

### Impact of tumor volume estimation on the population analysis of the Gompertz model

**Fig 4:**
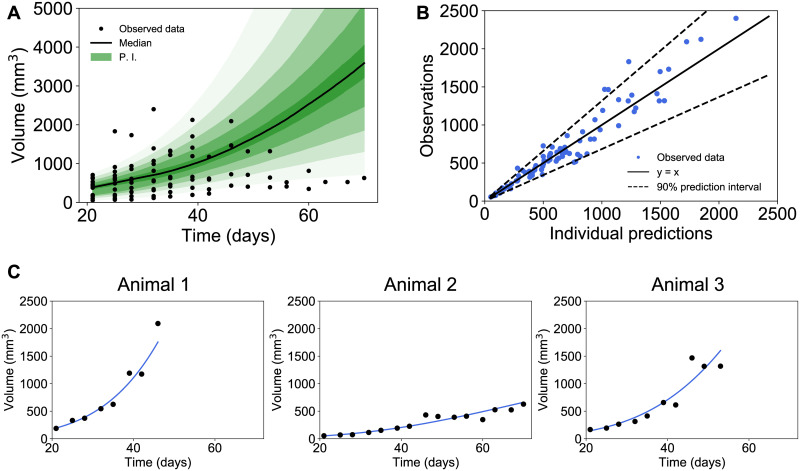
Population analysis of the Gompertz model fitted against data obtained with formula 2. (A) Prediction distribution of the Gompertz model fitted against the data. P.I. = prediction interval. (B) Observations vs individual predictions (C) Three representative examples of individual fits chosen randomly.

When trying to fit the same model to data obtained with different formulas, we found that formula 2 exhibited the best fits, together with formulas 1, and 3 (i.e., error model parameter *σ* was equal to 0.193, 0.193 and 0.194, respectively, S3 Table). Formulas 4, 5, 6 and 7 showed larger unexplained variability (*σ* = 0.218, 0.209, 0.216 and 0.218, respectively). Fig 4 shows the diagnostic plots relative to Formula 2 obtained with the population analysis. The prediction distribution (Fig 4A) was obtained from multiple simulations of all the individuals in the dataset, and it covered all individual data. The observations vs predictions (Fig 4B) showed that the distribution of the observations was symmetrically distributed around the predicted values and laid within the 90% prediction interval. These results suggested that the Gompertz model applied using Formula 2 could provide a good description of the dataset. Eventually, individual fits were accurate and confirmed these results (Figs 4C and S1). Good parametric identifiability was observed, as indicated by the low relative standard errors (R.S.E.) of all parameters. Specifically, all population-level parameters were identified with good precision (*α* = 0.235, *β* = 0.019 with R.S.E. of 3.12% and 8.14% for the two parameters, respectively, as shown in S3 Table). The coefficients of variation for *α* and *β* were estimated equal to 7.45% and 24.07%, respectively.

## Discussion

Improving ethic and tumor monitoring in mice has been a long-time goal for many research laboratories. Nowadays, many sophisticated imaging techniques have been developed to this matter, such as positron emission tomography (PET) or magnetic resonance (MR) that can even monitor tumor microenvironment [15,16]. However, because of cost, such techniques remain the exception and caliper measurement is the most common way to monitor tumors in xenografted mice.

In this study we outlined that 20% of experimental studies using caliper measurements did not mention the formula they used for tumor volume estimation. Such data are in line with the ARRIVE guidelines that previously reported a lack of key information in scientific publications [1]. We also outlined that there is a large variety of formulas, among which only two implement height dimension (i.e., Formula 1 and Formula 2). Indeed, because only a part of the tumor height is measurable, tumor’ heights are often underestimated and thus, not considered. However, we were not able to show a statistical difference between height measurement accuracy compared to length or width ones (i.e., MRE of dimensions before and after exeresis were of 0.121 ± 0.077, 0.144 ± 0.0862 and 0.214 ± 0.4828, for length, width and height, respectively, one-way ANOVA, p = 0.42). Of note, accuracy of formulas used with caliper for tumor growth monitoring, hasn’t been evaluated since 1989, when they recommended to focus on Formula 1 [17]. Thus, in this work we offered an update on the accuracy in volume prediction, using all formulas used in 2019 for breast cancer bearing mice.

To discriminate possible formula outliers, we first checked that they were all correlated to tumor weight (i.e., p < 0.05). Although a good correlation could mean a constant over- or under-estimation, it remains essential in comparative studies in tumor-bearing mice and thus should be considered. Secondly, we showed there was a strong statistical difference between formula’s MRE (one-way ANOVA, p = 2.10^−16^) suggesting ellipsoid type formulas (i.e., Formula 3, Formula 5 and Formula 4), that presented the lowest MRE (i.e., 0.272 ± 0.201, 0.275 ± 0.175 and 0.282 ± 0.192, respectively) were the most accurate. However, our further results on large tumor size population (i.e., 2008.4 ± 416.77 mg) showed that for tumors larger than 1968 ± 425 mg, these three formulas were no longer correlated to tumor weight. Indeed, this population was only well predicted with Formula 1 and 2 (Fig 3). Since Formula 1 and Formula 2 specificity is their implementation of height measurement, such results suggest the importance of this dimension, especially when working on large tumors. These results also confirmed Tomayko and Reynolds *et al*. conclusion, that size matters in tumor volume prediction [17].

Overall, to reduce animal experimentations and suffering, in the near future, we will need to use mathematical models more and more. Such *in silico* models could be used next in comparative studies, to evaluate differences in antiproliferative efficacy among treatments from a limited number of early observations. To have reliable models that can be used for *in silico* studies, they need themselves to rely on robust quantitative data that assess variabilities in tumor volume measurements, which we tested in our analysis. After establishing that all formulas were appropriate, we were able to use them in quantitative models such as Gompertz, known for its good descriptive and prediction performances in tumor growth [8–10]. Our results showed no statistical differences between formulas on model prediction, although better fits were observed for Formulas 2, 1 and 3 (i.e., error model parameter *σ* was equal to 0.193, 0.193 and 0.194, respectively, S3 Table). Of note, Gompertz analysis reaches the same limits than correlation analysis’ in terms of under- and over-estimation. However, the error model parameter (i.e., *σ*), not only includes the error of the model but measurements’ errors also, suggesting that better representation of real volume will be observed with lower *σ*.

**Fig 5:**
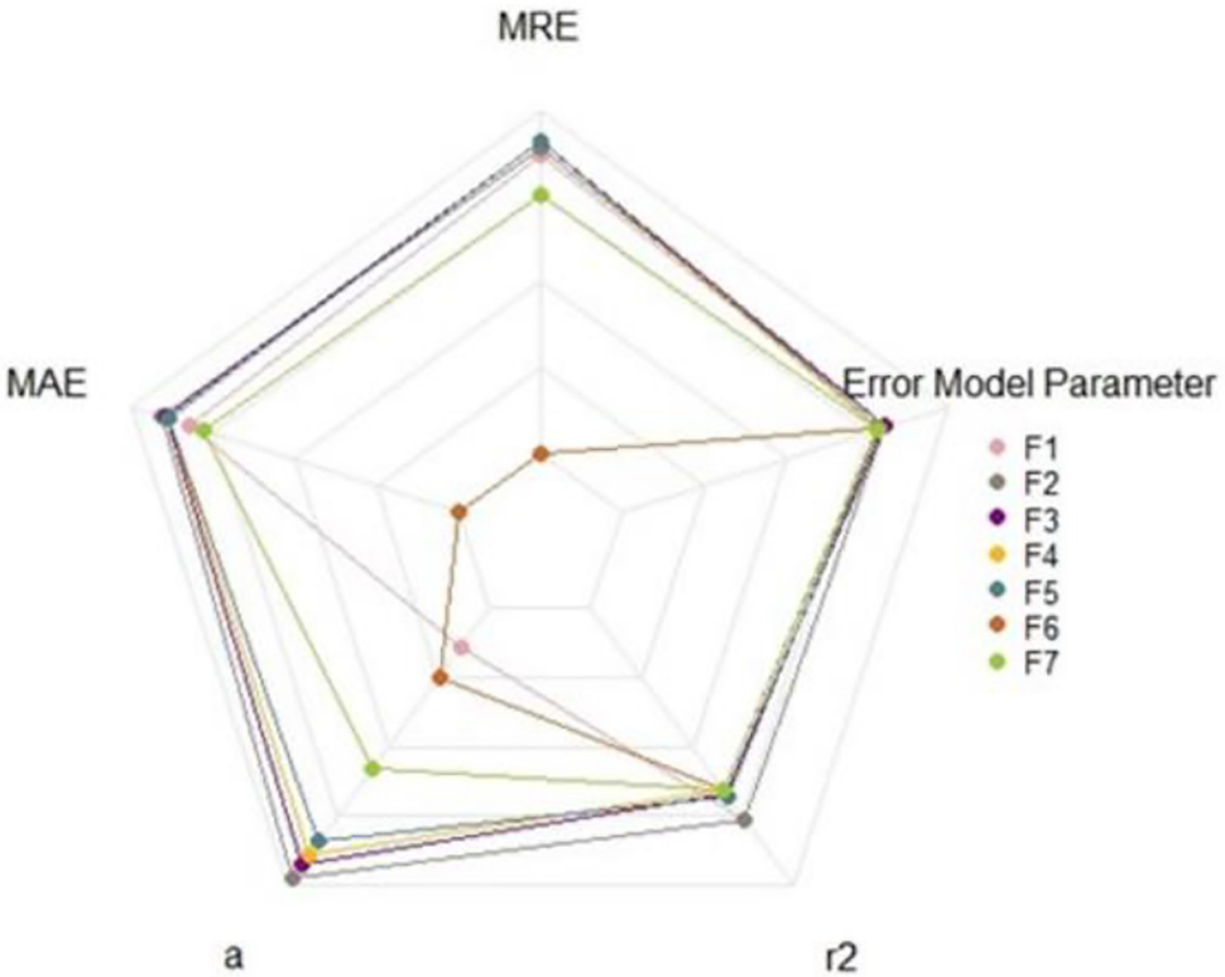
Radar chart displaying volume estimation data for Formula 1 (F1), Formula 2 (F2) Formula 3 (F3), Formula 4 (F4), Formula 5 (F5), Formula 6 (F6) and Formula 7 (F7). The larger the area, the better the prediction. All raw data used to make this chart are available in supplementary tables (S4 Table). *MRE = mean relative error, MAE = mean absolute error, r^2^ = correlation coefficient of regression line, a = slope of regression line.

Along these lines, correlation and Gompertz analysis should not be used as unique parameters for tumor volume estimation. Thus, when considering all results (i.e., MRE, r^2^, MAE, *a*, and error model parameter), we were able to exclude a few formulas that should no longer be used (i.e., Formula 6 and Formula 7). For the sake of standardization, we recommend researchers to focus on Formula 2 or Formula 3 (Fig 5). Based on our analysis on the impact of tumor size on tumor volume estimation we could even recommend using a hybrid strategy made of Formula 3 for slow growing tumor cells or tumors under 1500 mg and Formula 2 for larger tumors. Interestingly these formulas were only found in 0.5% of the studies (Table 1).

Of note, current ethical guidelines are evolving, and mice should no longer be sacrificed when reaching a tumor volume of 2000 mm^3^ but for a volume of 1500 mm^3^. Thus, Formula 3 only should be preferred for future tumor growth monitoring. In the past, Tomayko and Reynolds *et al*. recommended Formula 1 [17], but such difference could come from the fact that this was performed over 30 years ago, regulation has changed, thus, they probably analyzed larger tumors (i.e., 460 to 2200 mg), which we showed can have a strong impact on formula’s accuracy.

Finally, another solution here, could be to offer a correction of all existing formulas, using the equation of their respective regression line. Corrected formula would equal, *a* × *Formula* + *b* where *a* is the slope and *b* is the intercept of the regression line. Thus, lack of precision from tumor shape and underestimation of height dimension, could be corrected. For instance, using available data of S4 Table, Formula 1 would become 1.85439 × (1/6 π × L × W × H) − 23.944. However, this formula could be at risk of being dependent on the specific MDA MB 231 cell line analyzed in this study. Indeed, although this work is a proof of concept in formula standardization, other characteristics than size need to be investigated such as cell line, cancer type, treatment, mouse strain and engraftment localization.

## Supporting information

S1 Fig Individual fits obtained with the Gompertz model fitted against data obtained with formula 2. (DOCX)

S1 Table Summarize of the bibliographic search in the PubMed database from January 1^st^, 2019 to December 31^st^, 2019, using the followings terms: Xenograft, breast cancer, tumor growth and mice. * MR DWI = Diffusion-weighted magnetic resonance; PET CT = Positron emission tomography computing tomography; MD = missing data; NA = not applicable; unknown = information not accessible; Other = formulas did not describe a 3D representation.
(DOCX)

S2 Table Summarize of relevant animal data from protocol registration #2017031717108767 and #20181213155790720 (n = 87). * MD = Missing data.
(DOCX)

S3 Table Parameter estimates of the Gompertz model obtained fitting the data obtained with different formulations. *α*_0 is the specific growth rate (in 1/days), *β* is the parameter driving the exponential decrease of the proliferation rate (in 1/days), and *σ* is the error model parameter. R.S.E. is the relative standard error on parameter estimation. CV is the coefficient of variation expressed as 100∙exp(*ω*^2 − 1).
(DOCX)

S4 Table Volume estimation data (i.e., MRE, MAE, r^2^, *a* and Error Model Parameter) for Formula 1 (F1), Formula 2 (F2) Formula 3 (F3), Formula 4 (F4), Formula 5 (F5), Formula 6 (F6) and Formula 7 (F7). *MRE = mean relative error, MAE = mean absolute error, r^2^ = correlation coefficient of regression line, a = slope of regression line.
(DOCX)
