# Supplementary material for: Tumor growth monitoring in breast cancer xenografts: A good technique for a strong ethic
Source: PLoS One. 2022 Sep 30;17(9):e0274886. doi: 10.1371/journal.pone.0274886 (PMC9524649; doi:10.1371/journal.pone.0274886)
Supplement: S1 Fig — (DOCX) [file pone.0274886.s001.docx]

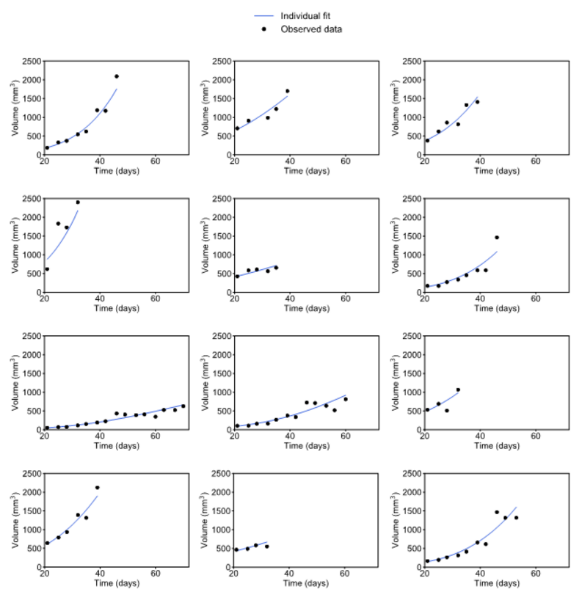


**S1 Fig:** **Individual fits obtained with the Gompertz model fitted against data obtained with formula 2.**
