# Supplementary material for: Tumor growth monitoring in breast cancer xenografts: A good technique for a strong ethic
Source: PLoS One. 2022 Sep 30;17(9):e0274886. doi: 10.1371/journal.pone.0274886 (PMC9524649; doi:10.1371/journal.pone.0274886)
Supplement: S1 Table — * MR DWI = Diffusion-weighted magnetic resonance; PET CT = Positron emission tomography computing tomography; MD = missing data; NA = not applicable; unknown = information not accessible; Other = formulas did not describe a 3D representation. (DOCX) [file pone.0274886.s002.docx]

|  | References | Method | Formula |
| --- | --- | --- | --- |
| 1 | Mol Immunol. 2020 Feb;118:174-181 | caliper | 1 |
| 2 | Int J Radiat Oncol Biol Phys. 2019 Oct 10:S0360-3016(19)33868-4. | caliper | 1 |
| 3 | [Int J Mol Sci. 2019 Aug 8;20(16):3873](https://www-ncbi-nlm-nih-gov.proxy.insermbiblio.inist.fr/pmc/articles/PMC6719192/) | caliper | 1 |
| 4 | Phytomedicine. Volume 62, September 2019, 152962 | caliper | 1 |
| 5 | [Oncotarget. 2019 Mar 8; 10(20): 1887–1902.](https://www-ncbi-nlm-nih-gov.proxy.insermbiblio.inist.fr/pmc/articles/PMC6443012/) | caliper | 1 |
| 6 | [Cancers (Basel). 2019 Apr; 11(4): 460.](https://www-ncbi-nlm-nih-gov.proxy.insermbiblio.inist.fr/pmc/articles/PMC6521103/) | caliper | 1 |
| 7 | [J Immunother Cancer. 2019; 7: 29.](https://www-ncbi-nlm-nih-gov.proxy.insermbiblio.inist.fr/pmc/articles/PMC6360707/) | caliper | 1 |
| 8 | [Yonsei Med J. 2019 Feb 1; 60(2): 148–157.](https://www-ncbi-nlm-nih-gov.proxy.insermbiblio.inist.fr/pmc/articles/PMC6342713/) | caliper | 1 |
| 9 | [Cancer Gene Therapy volume 26, pages374–387(2019)](https://www-nature-com.proxy.insermbiblio.inist.fr/cgt) | caliper | 1 |
| 10 | Colloids Surf B Biointerfaces. 2019 Feb 1;174:80-86 | caliper | 1 |
| 11 | J Steroid Biochem Mol Biol. 2019 Oct;193:105415 | caliper | 1 |
| 12 | J Nat Prod. 2019 Nov 22;82(11):3140-3149 | caliper | 1 |
| 13 | [Cancer Lett. 2019 May 1; 449: 66–75.](https://www-ncbi-nlm-nih-gov.proxy.insermbiblio.inist.fr/entrez/eutils/elink.fcgi?dbfrom=pubmed&retmode=ref&cmd=prlinks&id=30771431) | caliper | 2 |
| 14 | Cancer Letters. Volume 449, 1 May 2019, Pages 196-206 | caliper | 3 |
| 15 | [Aging (Albany NY). 2019 Dec 15; 11(23): 11054–11072.](https://www-ncbi-nlm-nih-gov.proxy.insermbiblio.inist.fr/pmc/articles/PMC6932924/) | caliper | 4 |
| 16 | [Oncotarget. 2019 Nov 12; 10(61): 6561–6576.](https://www-ncbi-nlm-nih-gov.proxy.insermbiblio.inist.fr/pmc/articles/PMC6859925/) | caliper | 4 |
| 17 | [Int J Mol Sci. 2019 Nov; 20(22): 5626.](https://www-ncbi-nlm-nih-gov.proxy.insermbiblio.inist.fr/pmc/articles/PMC6888424/) | caliper | 4 |
| 18 | Colloids and Surfaces B: Biointerfaces. Volume 185, 1 January 2020, 110585 | caliper | 4 |
| 19 | [Front Pharmacol. 2019; 10: 1195.](https://www-ncbi-nlm-nih-gov.proxy.insermbiblio.inist.fr/pmc/articles/PMC6796319/) | caliper | 4 |
| 20 | [Int J Mol Sci. 2019 Oct; 20(19): 4763.](https://www-ncbi-nlm-nih-gov.proxy.insermbiblio.inist.fr/pmc/articles/PMC6801585/) | caliper | 4 |
| 21 | [Int J Nanomedicine. 2019; 14: 6971–6988.](https://www-ncbi-nlm-nih-gov.proxy.insermbiblio.inist.fr/pmc/articles/PMC6718740/) | caliper | 4 |
| 22 | [Molecules. 2019 Sep; 24(18): 3241.](https://www-ncbi-nlm-nih-gov.proxy.insermbiblio.inist.fr/pmc/articles/PMC6767168/) | caliper | 4 |
| 23 | [J Exp Clin Cancer Res. 2019; 38: 388.](https://www-ncbi-nlm-nih-gov.proxy.insermbiblio.inist.fr/pmc/articles/PMC6727545/) | caliper | 4 |
| 24 | Cancer Letters Volume 465, 28 November 2019, Pages 12-23 | caliper | 4 |
| 25 | [J Exp Clin Cancer Res. 2019; 38: 383.](https://www-ncbi-nlm-nih-gov.proxy.insermbiblio.inist.fr/pmc/articles/PMC6717344/) | caliper | 4 |
| 26 | Stem Cell Res. 2019 Oct;40:101538 | caliper | 4 |
| 27 | Cancer Res October 15 2019 (79) (20) 5233-5244 | caliper | 4 |
| 28 | Pharmacol Res. 2019 Sep;147:104387 | caliper | 4 |
| 29 | Nanomedicine. 2019 Oct;21:102066 | caliper | 4 |
| 30 | Mol Cancer Ther 2019 Oct;18(10):1755-1764 | caliper | 4 |
| 31 | J Pharm Biomed Anal. 2019 Sep 10;174:718-727 | caliper | 4 |
| 32 | [BMC Cancer. 2019; 19: 642.](https://www-ncbi-nlm-nih-gov.proxy.insermbiblio.inist.fr/pmc/articles/PMC6599331/) | caliper | 4 |
| 33 | [Cancer Res. 2019 Aug 15; 79(16): 4211–4226.](https://www-ncbi-nlm-nih-gov.proxy.insermbiblio.inist.fr/entrez/eutils/elink.fcgi?dbfrom=pubmed&retmode=ref&cmd=prlinks&id=31239273) | caliper | 4 |
| 34 | Carbohydr Polym. 2019 Oct 1;221:84-93 | caliper | 4 |
| 35 | [Front Oncol. 2019; 9: 461.](https://www-ncbi-nlm-nih-gov.proxy.insermbiblio.inist.fr/pmc/articles/PMC6558049/) | caliper | 4 |
| 36 | [Cancer Manag Res. 2019; 11: 5075–5089.](https://www-ncbi-nlm-nih-gov.proxy.insermbiblio.inist.fr/pmc/articles/PMC6549700/) | caliper | 4 |
| 37 | [Molecules. 2019 Jun; 24(12): 2255.](https://www-ncbi-nlm-nih-gov.proxy.insermbiblio.inist.fr/pmc/articles/PMC6631792/) | caliper | 4 |
| 38 | [J Exp Clin Cancer Res. 2019; 38: 248.](https://www-ncbi-nlm-nih-gov.proxy.insermbiblio.inist.fr/pmc/articles/PMC6558706/) | caliper | 4 |
| 39 | [BMC Cancer. 2019; 19: 555.](https://www-ncbi-nlm-nih-gov.proxy.insermbiblio.inist.fr/pmc/articles/PMC6558813/) | caliper | 4 |
| 40 | J Biotechnol. 2019 Jul 20;300:70-77 | caliper | 4 |
| 41 | [Cell Death Dis. 2019 Jun; 10(6): 423.](https://www-ncbi-nlm-nih-gov.proxy.insermbiblio.inist.fr/pmc/articles/PMC6541642/) | caliper | 4 |
| 42 | Cellular physiology. Volume234, Issue12 | caliper | 4 |
| 43 | Eur J Pharmacol. 2019 Aug 5;856:172407 | caliper | 4 |
| 44 | [Theranostics. 2019; 9(9): 2618–2636.](https://www-ncbi-nlm-nih-gov.proxy.insermbiblio.inist.fr/pmc/articles/PMC6525995/) | caliper | 4 |
| 45 | [Redox Biol. 2019 May; 23: 101175.](https://www-ncbi-nlm-nih-gov.proxy.insermbiblio.inist.fr/pmc/articles/PMC6859578/) | caliper | 4 |
| 46 | Hum Gene Ther. 2019 Sep;30(9):1117-1132 | caliper | [4](https://www-liebertpub-com.proxy.insermbiblio.inist.fr/doi/10.1089/hum.2019.059#B2) |
| 47 | [J Exp Clin Cancer Res. 2019 May 22;38(1):211](https://www-ncbi-nlm-nih-gov.proxy.insermbiblio.inist.fr/pmc/articles/PMC6532146/) | caliper | 4 |
| 48 | Breast Cancer Res. 2019 May 14;21(1):61 | caliper | 4 |
| 49 | Mol Ther. 2019 Jul 3;27(7):1252-1261 | caliper | 4 |
| 50 | [Quant Imaging Med Surg. 2019 Mar; 9(3): 418–426.](https://www-ncbi-nlm-nih-gov.proxy.insermbiblio.inist.fr/pmc/articles/PMC6462576/) | caliper | 4 |
| 51 | [In Vivo. 2019 May-Jun; 33(3): 821–825.](https://www-ncbi-nlm-nih-gov.proxy.insermbiblio.inist.fr/pmc/articles/PMC6559888/) | caliper | 4 |
| 52 | Maturitas Volume 123, May 2019, Pages 1-8 | caliper | 4 |
| 53 | [Oxid Med Cell Longev. 2019 Mar 24;2019:9296439](https://www-ncbi-nlm-nih-gov.proxy.insermbiblio.inist.fr/pmc/articles/PMC6451810/) | caliper | 4 |
| 54 | *Carcinogenesis*, Volume 40, Issue 12, December 2019, Pages 1469–1479 | caliper | 4 |
| 55 | [Oncogene volume 38, pages5551–5565(2019)](https://www-nature-com.proxy.insermbiblio.inist.fr/onc) | caliper | 4 |
| 56 | [Cancer Sci. 2019 Jun; 110(6): 1909–1920.](https://www-ncbi-nlm-nih-gov.proxy.insermbiblio.inist.fr/pmc/articles/PMC6549921/) | caliper | 4 |
| 57 | [J Thromb Haemost. 2019 Jun; 17(6): 951–963.](https://www-ncbi-nlm-nih-gov.proxy.insermbiblio.inist.fr/pmc/articles/PMC6849835/) | caliper | 4 |
| 58 | Life Sciences Volume 224, 1 May 2019, Pages 204-211 | caliper | 4 |
| 59 | [Mol Cancer. 2019 Mar 28;18(1):46](https://www-ncbi-nlm-nih-gov.proxy.insermbiblio.inist.fr/pmc/articles/PMC6437932/) | caliper | 4 |
| 60 | Mol Cancer Ther. 2019 May;18(5):873-885 | caliper | 4 |
| 61 | [Theranostics. 2019; 9(3): 761–777.](https://www-ncbi-nlm-nih-gov.proxy.insermbiblio.inist.fr/pmc/articles/PMC6376470/) | caliper | 4 |
| 62 | Phytomedicine Volume 57, April 2019, Pages 245-254 | caliper | 4 |
| 63 | [J Exp Clin Cancer Res. 2019; 38: 131.](https://www-ncbi-nlm-nih-gov.proxy.insermbiblio.inist.fr/pmc/articles/PMC6423756/) | caliper | 4 |
| 64 | [Cell Death Dis. 2019 Mar; 10(3): 248.](https://www-ncbi-nlm-nih-gov.proxy.insermbiblio.inist.fr/pmc/articles/PMC6416354/) | caliper | 4 |
| 65 | [ACS Appl Mater Interfaces. 2019 Apr 3; 11(13): 12342–12356.](https://www-ncbi-nlm-nih-gov.proxy.insermbiblio.inist.fr/entrez/eutils/elink.fcgi?dbfrom=pubmed&retmode=ref&cmd=prlinks&id=30860347) | caliper | 4 |
| 66 | [Toxicology Volume 418, 15 April 2019, Pages 22-31](https://www-sciencedirect-com.proxy.insermbiblio.inist.fr/science/journal/0300483X) | caliper | 4 |
| 67 | [J Hematol Oncol. 2019; 12: 19.](https://www-ncbi-nlm-nih-gov.proxy.insermbiblio.inist.fr/pmc/articles/PMC6387548/) | caliper | 4 |
| 68 | Cancer Letters Volume 449, 1 May 2019, Pages 45-55 | caliper | 4 |
| 69 | International Journal of Pharmaceutics Volume 560, 5 April 2019, Pages 191-204 | caliper | 4 |
| 70 | Pharmacol Res. 2019 Apr;142:1-13 | caliper | 4 |
| 71 | Cancer Lett. 2019 Apr 10;447:141-153. | caliper | 4 |
| 72 | [Sci Rep. 2019; 9: 966.](https://www-ncbi-nlm-nih-gov.proxy.insermbiblio.inist.fr/pmc/articles/PMC6353949/) | caliper | 4 |
| 73 | [Clin Cancer Res. 2019 May 1; 25(9): 2725–2736.](https://www-ncbi-nlm-nih-gov.proxy.insermbiblio.inist.fr/entrez/eutils/elink.fcgi?dbfrom=pubmed&retmode=ref&cmd=prlinks&id=30635338) | caliper | 4 |
| 74 | [Cancer Res. 2019 May 1; 79(9): 2152–2166.](https://www-ncbi-nlm-nih-gov.proxy.insermbiblio.inist.fr/entrez/eutils/elink.fcgi?dbfrom=pubmed&retmode=ref&cmd=prlinks&id=30635277) | caliper | 4 |
| 75 | Biochem Pharmacol. 2019 Mar;161:73-88 | caliper | 4 |
| 76 | Acta Biomaterialia Volume 86, 1 March 2019, Pages 280-290 | caliper | 4 |
| 77 | [Breast Cancer Res Treat. 2019 Apr; 174(3): 615–625.](https://www-ncbi-nlm-nih-gov.proxy.insermbiblio.inist.fr/entrez/eutils/elink.fcgi?dbfrom=pubmed&retmode=ref&cmd=prlinks&id=30607633) | caliper | 4 |
| 78 | Int J Pharm. 2019 Feb 10;556:263-275 | caliper | 4 |
| 79 | Mol Carcinog. 2019 Apr;58(4):461-473. | caliper | 4 |
| 80 | Mol Cancer Res. 2019 Feb; 17(2): 604–617. | caliper | 4 |
| 81 | Clin Cancer Res. 2019 Jan 1;25(1):414-425 | caliper | 4 |
| 82 | [Biosci Rep. 2019 Feb 28; 39(2): BSR20180480.](https://www-ncbi-nlm-nih-gov.proxy.insermbiblio.inist.fr/pmc/articles/PMC6361774/) | caliper | 4 |
| 83 | Int J Cancer. 2019 Feb 1;144(3):615-630 | caliper | 4 |
| 84 | [J Natl Cancer Inst. 2019 Apr; 111(4): 388–398.](https://www-ncbi-nlm-nih-gov.proxy.insermbiblio.inist.fr/pmc/articles/PMC6449168/) | caliper | 4 |
| 85 | [Acta Pharmacol Sin. 2019 Apr; 40(4): 530–538.](https://www-ncbi-nlm-nih-gov.proxy.insermbiblio.inist.fr/pmc/articles/PMC6462016/) | caliper | 4 |
| 86 | [Acta Pharmacol Sin. 2019 Feb; 40(2): 243–256.](https://www-ncbi-nlm-nih-gov.proxy.insermbiblio.inist.fr/pmc/articles/PMC6329750/) | caliper | 4 |
| 87 | *Oncoimmunology. 2019; 8(11): 1641392.* | caliper | 4 |
| 88 | J Neurooncol. 2019 Nov;145(2):211-222. | caliper | 4 |
| 89 | Bioengineered. 2019 Dec;10(1):374-382 | caliper | 4 |
| 90 | Histochem Cell Biol. 2019 Oct;152(4):281-291 | caliper | 4 |
| 91 | Breast Cancer Res Treat. 2019 Sep;177(2):357-367 | caliper | 4 |
| 92 | Biol Pharm Bull. 2019 Jun 1;42(6):892-899 | caliper | 4 |
| 93 | [Chin J Integr Med. 2019 Jun;25(6):425-430](https://link-springer-com.proxy.insermbiblio.inist.fr/journal/11655) | caliper | 4 |
| 94 | J Tradit Chin Med 2019 October 15; 39(5): 642-648 | caliper | 4 |
| 95 | [Front Oncol. 2019; 9: 1534.](https://www.ncbi.nlm.nih.gov/pmc/articles/PMC6989603/) | caliper | 4 |
| 96 | Biomed Pharmacother. 2020 Mar;123:109616 | caliper | 4 |
| 97 | Mol Cancer Ther. 2020 Mar;19(3):868-881 | caliper | 4 |
| 98 | Breast Cancer Res. 2019 Dec 18;21(1):146 | caliper | 4 |
| 99 | Pathol Res Pract. 2020 Feb;216(2):152772 | caliper | 4 |
| 100 | Int J Pharm. 2020 Jan 25;574:118939 | caliper | 4 |
| 101 | Nanomedicine. 2020 Feb;24:102124 | caliper | 4 |
| 102 | J Neuroimmune Pharmacol. 2020 Sep;15(3):487-500 | caliper | 4 |
| 103 | Cancer Lett. 2020 Jul 10;482:102-111 | caliper | 4 |
| 104 | Cancer Res. 2020 Feb 1;80(3):418-429 | caliper | 4 |
| 105 | J Cell Physiol. 2020 May;235(5):4814-4823 | caliper | 4 |
| 106 | Mol Cancer Ther. 2020 Feb;19(2):348-363 | caliper | 4 |
| 107 | Clin Cancer Res. 2019 Dec 1;25(23):7229-7242 | caliper | 4 |
| 108 | Nanomedicine (Lond). 2019 Sep;14(17):2315-2338 | caliper | 4 |
| 109 | Cell Death Differ. 2020 Mar;27(3):1105-1118 | caliper | 4 |
| 110 | J Mater Chem B. 2019 Oct 16;7(40):6075-6086 | caliper | 4 |
| 111 | J Cell Physiol. 2020 Feb;235(2):932-943 | caliper | 4 |
| 112 | Cancer Med. 2019 Aug;8(9):4389-4403 | caliper | 4 |
| 113 | Vaccine. 2019 Jul 18;37(31):4382-4391 | caliper | 4 |
| 114 | Front Immunol. 2019 May 24;10:1149 | caliper | 4 |
| 115 | J Cell Physiol. 2019 Dec;234(12):23409-23420 | caliper | 4 |
| 116 | Sci Rep. 2019 May 17;9(1):7509 | caliper | 4 |
| 117 | JCI Insight. 2019 Mar 21;5(9):e125094 | caliper | 4 |
| 118 | [Sci Rep. 2019; 9: 4802.](https://www.ncbi.nlm.nih.gov/pmc/articles/PMC6423038/) | caliper | 4 |
| 119 | Breast Cancer Res Treat. 2019 Jun;175(3):553-566 | caliper | 4 |
| 120 | Clin Cancer Res. 2019 Jun 15;25(12):3658-3672 | caliper | 4 |
| 121 | Mol Cancer Res. 2019 Feb;17(2):409-419 | caliper | 4 |
| 122 | Hum Gene Ther. 2019 Feb;30(2):197-210 | caliper | 4 |
| 123 | Pharmacol Res. 2019 Dec;150:104517 | caliper | 5 |
| 124 | Toxicol Appl Pharmacol. 2019 Dec 1;384:114789 | caliper | 5 |
| 125 | Artif Cells Nanomed Biotechnol. 2019 Dec;47(1):4001-4011. | caliper | 5 |
| 126 | [PLoS One. 2019; 14(9): e0222580.](https://www-ncbi-nlm-nih-gov.proxy.insermbiblio.inist.fr/pmc/articles/PMC6752870/) | caliper | 5 |
| 127 | [Breast Cancer Res. 2019; 21: 98.](https://www-ncbi-nlm-nih-gov.proxy.insermbiblio.inist.fr/pmc/articles/PMC6714238/) | caliper | 5 |
| 128 | Exp Clin Cancer Res . 2019 Aug 19;38(1):363 | caliper | 5 |
| 129 | [Cells. 2019 Jul; 8(7): 750.](https://www-ncbi-nlm-nih-gov.proxy.insermbiblio.inist.fr/pmc/articles/PMC6678306/) | caliper | 5 |
| 130 | [J Exp Clin Cancer Res. 2019; 38: 306](https://www-ncbi-nlm-nih-gov.proxy.insermbiblio.inist.fr/pmc/articles/PMC6626398/) | caliper | 5 |
| 131 | Mol Pharm. 2019 Aug 5;16(8):3477-3488 | caliper | 5 |
| 132 | Eur J Med Chem. 2019 Aug 15;176:393-409 | caliper | 5 |
| 133 | [Oncotarget. 2019 Mar 22; 10(24): 2355–2368.](https://www-ncbi-nlm-nih-gov.proxy.insermbiblio.inist.fr/pmc/articles/PMC6481325/) | caliper | 5 |
| 134 | Exp Cell Res. 2019 Jul 15;380(2):141-148 | caliper | 5 |
| 135 | Acta Biomaterialia Volume 88, 1 April 2019, Pages 448-461 | caliper | 5 |
| 136 | Cancer Res. 2019 May 15;79(10):2669-2683 | caliper | 5 |
| 137 | [BMC Cancer. 2019; 19: 234.](https://www-ncbi-nlm-nih-gov.proxy.insermbiblio.inist.fr/pmc/articles/PMC6419843/) | caliper | 5 |
| 138 | [Drug Des Devel Ther. 2019; 13: 757–766.](https://www-ncbi-nlm-nih-gov.proxy.insermbiblio.inist.fr/pmc/articles/PMC6391152/) | caliper | 5 |
| 139 | [BMC Cancer. 2019; 19: 211.](https://www-ncbi-nlm-nih-gov.proxy.insermbiblio.inist.fr/pmc/articles/PMC6408845/) | caliper | 5 |
| 140 | [J Exp Clin Cancer Res. 2019; 38: 94.](https://www-ncbi-nlm-nih-gov.proxy.insermbiblio.inist.fr/pmc/articles/PMC6385430/) | caliper | 5 |
| 141 | [World J Nucl Med. 2019 Jan-Mar; 18(1): 18–24.](https://www-ncbi-nlm-nih-gov.proxy.insermbiblio.inist.fr/pmc/articles/PMC6357706/) | caliper | 5 |
| 142 | Acta Biomater. 2019 Jan 15;84:367-377 | caliper | 5 |
| 143 | ACS Appl Mater Interfaces. 2019 Nov 20;11(46):42873-42884. | caliper | 5 |
| 144 | [Cancer Chemotherapy and Pharmacology volume 84, pages1241–1256(2019)](https://link-springer-com.proxy.insermbiblio.inist.fr/journal/280) | caliper | 5 |
| 145 | [Breast Cancer Res Treat. 2019 Aug;177(1):29-40](https://link-springer-com.proxy.insermbiblio.inist.fr/journal/10549) | caliper | 5 |
| 146 | DNA Cell Biol. 2019 Jun;38(6):501-509. | caliper | 5 |
| 147 | Oncol Rep. 2019 Apr;41(4):2418-2430 | caliper | 5 |
| 148 | FASEB J. 2019 Jun;33(6):7301-7314. | caliper | 5 |
| 149 | J Med Chem. 2019 Nov 27;62(22):10204-10220 | caliper | 5 |
| 150 | Biomacromolecules. 2020 Jan 13;21(1):104-113 | caliper | 5 |
| 151 | Clin Cancer Res. 2019 Sep 15;25(18):5702-5716 | caliper | 5 |
| 152 | Toxicol Sci. 2019 Aug 1;170(2):283-295 | caliper | 5 |
| 153 | EBioMedicine. 2019 Feb;40:118-134 | caliper | 5 |
| 154 | J Mater Chem B. 2019 Jan 28;7(4):576-585 | caliper | 5 |
| 155 | Int J Cancer. 2019 Feb 1;144(3):651-664 | caliper | 5 |
| 156 | Lasers Surg Med. 2019 Apr;51(4):352-362 | caliper | 5 |
| 157 | Nitric Oxide. Volume 93, 1 December 2019, Pages 34-43 | caliper | 6 |
| 158 | Int J Radiat Oncol Biol Phys. 2020 Sep 1;108(1):115-125 | caliper | 6 |
| 159 | Theranostics. 2019 Jun 24;9(16):4608-4623 | caliper | 7 |
| 160 | Antioxid Redox Signal. 2019 Jun 10;30(17):1983-1998 | caliper | 7 |
| 161 | [Breast Cancer. 2020 Jan;27(1):147-158](https://www-ncbi-nlm-nih-gov.proxy.insermbiblio.inist.fr/pmc/articles/PMC6921513/) | caliper | MD |
| 162 | [J Biol Methods. 2019; 6(4): e121.](https://www-ncbi-nlm-nih-gov.proxy.insermbiblio.inist.fr/pmc/articles/PMC6974696/) | caliper | MD |
| 163 | [Int J Med Sci. 2019; 16(12): 1642–1651.](https://www-ncbi-nlm-nih-gov.proxy.insermbiblio.inist.fr/pmc/articles/PMC6909807/) | caliper | MD |
| 164 | [Dis Markers. 2019; 2019: 8186091.](https://www-ncbi-nlm-nih-gov.proxy.insermbiblio.inist.fr/pmc/articles/PMC6886328/) | caliper | MD |
| 165 | [Drug Deliv. 2019; 26(1): 1254–1264.](https://www-ncbi-nlm-nih-gov.proxy.insermbiblio.inist.fr/pmc/articles/PMC6882488/) | caliper | MD |
| 166 | Front Pharmacol. 2019; 10: 1202. | caliper | MD |
| 167 | Int J Nanomedicine. 2019 Oct 9;14:8073-8094. | caliper | MD |
| 168 | Cancer Letters Volume 465, 28 November 2019, Pages 68-81 | caliper | MD |
| 169 | [Int J Cancer. 2020 Mar 15; 146(6): 1674–1685.](https://www-ncbi-nlm-nih-gov.proxy.insermbiblio.inist.fr/pmc/articles/PMC7003894/) | caliper | MD |
| 170 | Sci Rep. 2019 Jun 24;9(1):9204. | caliper | MD |
| 171 | [Nanotheranostics. 2019; 3(2): 212–222.](https://www-ncbi-nlm-nih-gov.proxy.insermbiblio.inist.fr/pmc/articles/PMC6536781/) | caliper | MD |
| 172 | [J Cancer Res Clin Oncol. 2019; 145(8): 1999–2012.](https://www-ncbi-nlm-nih-gov.proxy.insermbiblio.inist.fr/pmc/articles/PMC6658578/) | caliper | MD |
| 173 | \| Cancer Res. 2019 Aug 15;79(16):4042-4056 \| \| --- \| | caliper | MD |
| 174 | [J Exp Clin Cancer Res. 2019; 38: 225.](https://www-ncbi-nlm-nih-gov.proxy.insermbiblio.inist.fr/pmc/articles/PMC6540563/) | caliper | MD |
| 175 | [Neoplasia. 2019 Jul; 21(7): 627–640.](https://www-ncbi-nlm-nih-gov.proxy.insermbiblio.inist.fr/pmc/articles/PMC6520639/) | caliper | MD |
| 176 | Molecular Cancer Biology Volume145, Issue10 15 November 2019 Pages 2767-2780 | caliper | MD |
| 177 | [Stem Cell Res Ther. 2019 Apr 11;10(1):117](https://www-ncbi-nlm-nih-gov.proxy.insermbiblio.inist.fr/pmc/articles/PMC6458638/) | caliper | MD |
| 178 | [J Exp Clin Cancer Res. 2019; 38: 134.](https://www-ncbi-nlm-nih-gov.proxy.insermbiblio.inist.fr/pmc/articles/PMC6429712/) | caliper | MD |
| 179 | Int J Mol Sci. 2019 Mar 20;20(6):1419. | caliper | MD |
| 180 | [Breast Cancer Res. 2019 Feb 18;21(1):27](https://www-ncbi-nlm-nih-gov.proxy.insermbiblio.inist.fr/pmc/articles/PMC6380056/) | caliper | MD |
| 181 | [J Immunother Cancer. 2019 Feb 8;7(1):37](https://www-ncbi-nlm-nih-gov.proxy.insermbiblio.inist.fr/pmc/articles/PMC6368764/) | caliper | MD |
| 182 | macromolecular bioscience Volume19, Issue4 April 2019 1800445 | caliper | MD |
| 183 | [Cancer Med. 2019 Jan; 8(1): 325–336.](https://www-ncbi-nlm-nih-gov.proxy.insermbiblio.inist.fr/pmc/articles/PMC6346259/) | caliper | MD |
| 184 | [Oncogene. 2019 Mar; 38(12): 2123–2134.](https://www-ncbi-nlm-nih-gov.proxy.insermbiblio.inist.fr/entrez/eutils/elink.fcgi?dbfrom=pubmed&retmode=ref&cmd=prlinks&id=30455428) | caliper | MD |
| 185 | [Int J Cancer. 2019 Apr 15; 144(8): 1996–2007.](https://www-ncbi-nlm-nih-gov.proxy.insermbiblio.inist.fr/pmc/articles/PMC6590135/) | caliper | MD |
| 186 | Chem Biol Drug Des. 2019 Mar;93(3):232-241 | caliper | MD |
| 187 | *J Natl Cancer Inst. 2019 Apr 1;111(4):399-408* | caliper | MD |
| 188 | J Cell Physiol. 2019 Mar;234(3):2880-2894 | caliper | MD |
| 189 | [Oncogene. 2019 Jan; 38(3): 390–405.](https://www-ncbi-nlm-nih-gov.proxy.insermbiblio.inist.fr/entrez/eutils/elink.fcgi?dbfrom=pubmed&retmode=ref&cmd=prlinks&id=30111819) | caliper | MD |
| 190 | [Cell Death Differ. 2019 May; 26(5): 812–825](https://www-ncbi-nlm-nih-gov.proxy.insermbiblio.inist.fr/pmc/articles/PMC6461859/) | caliper | MD |
| 191 | Breast Cancer Res Treat. 2019 Sep;177(2):307-323 | caliper | MD |
| 192 | Neoplasma. 2019 Jun 3;66(5):746-755. | caliper | MD |
| 193 | Nanoscale. 2019 Jun 20;11(24):11470-11483. | caliper | MD |
| 194 | [J Cancer Res Clin Oncol. 2019 May;145(5):1191-1200](https://link-springer-com.proxy.insermbiblio.inist.fr/journal/432) | caliper | MD |
| 195 | Oncol Rep. 2019 Jan;41(1):455-464. | caliper | MD |
| 196 | FASEB J. 2020 Jan;34(1):1447-1464 | caliper | MD |
| 197 | Cancer Biol Ther. 2020;21(2):178-188 | caliper | MD |
| 198 | Eur Rev Med Pharmacol Sci. 2019 Sep;23(17):7457-7468 | caliper | MD |
| 199 | Mol Cancer Ther. 2020 Jan;19(1):13-25 | caliper | MD |
| 200 | [Cancer Cell Int. 2019; 19: 75](https://www.ncbi.nlm.nih.gov/pmc/articles/PMC6441222/) | caliper | MD |
| 201 | Int J Cancer. 2019 Oct 1;145(7):1874-1888 | caliper | MD |
| 202 | Life Sci. 2019 Feb 1;218:16-24 | caliper | MD |
| 203 | [Aesthetic Plast Surg. 2019 Apr;43(2):498-513](https://link-springer-com.proxy.insermbiblio.inist.fr/journal/266) | bioluminescence_imaging | NA |
| 204 | [Cancer Immunol Immunother. 2020; 69(2): 163–174.](https://www-ncbi-nlm-nih-gov.proxy.insermbiblio.inist.fr/pmc/articles/PMC7000514/) | bioluminescence_imaging | NA |
| 205 | Mol Cell. 2019 Sep 5;75(5):967-981.e9 | bioluminescence_imaging | NA |
| 206 | [J Transl Med. 2019; 17: 201.](https://www-ncbi-nlm-nih-gov.proxy.insermbiblio.inist.fr/pmc/articles/PMC6582486/) | bioluminescence_imaging | NA |
| 207 | [Oncol Rep. 2019 Apr; 41(4): 2126–2136.](https://www-ncbi-nlm-nih-gov.proxy.insermbiblio.inist.fr/pmc/articles/PMC6412463/) | bioluminescence_imaging | NA |
| 208 | [Sci Adv. 2019 Feb; 5(2): eaav5590.](https://www-ncbi-nlm-nih-gov.proxy.insermbiblio.inist.fr/pmc/articles/PMC6365116/) | bioluminescence_imaging | NA |
| 209 | Cancer Letters Volume 447, 10 April 2019, Pages 24-32 | bioluminescence_imaging | NA |
| 210 | Biomaterials Volumes 190–191, January 2019, Pages 38-50 | bioluminescence_imaging | NA |
| 211 | Mol Cancer Ther. 2020 Mar;19(3):802-811. | bioluminescence_imaging | NA |
| 212 | Clin Cancer Res. 2019 Aug 1;25(15):4846-4858 | bioluminescence_imaging | NA |
| 213 | Clin Cancer Res. 2019 May 1;25(9):2769-2782 | bioluminescence_imaging | NA |
| 214 | [Theranostics. 2019; 9(4): 1154–1169.](https://www-ncbi-nlm-nih-gov.proxy.insermbiblio.inist.fr/pmc/articles/PMC6401411/) | fluorescence_imaging | NA |
| 215 | Translational Cancer Mechanisms and Therapy May 2019 Volume 25, Issue 9 | fluorescence_imaging | NA |
| 216 | J Med Chem. 2019 Feb 28;62(4):2172-2183 | fluorescence_imaging | NA |
| 217 | [Cancer Med. 2019 Apr; 8(4): 1594–1603.](https://www-ncbi-nlm-nih-gov.proxy.insermbiblio.inist.fr/pmc/articles/PMC6488150/) | mr_dwi_imaging | NA |
| 218 | J Biomed Nanotechnol. 2019 Nov 1;15(11):2229-2239 | NA | NA |
| 219 | Biol Pharm Bull. 2019;42(11):1921-1925. | NA | NA |
| 220 | *Biochemistry and Biotechnology* Volume: 190 Issue 3 (2020) | NA | NA |
| 221 | Cell Cycle. 2019 Nov;18(21):2914-2927. | NA | NA |
| 222 | ACS Appl Mater Interfaces. 2019 Sep 11;11(36):32670-32678. | NA | NA |
| 223 | Curr Pharm Des. 2019;25(8):862-870. | NA | NA |
| 224 | Cell Physiol Biochem. 2019;52(3):382-396. | NA | NA |
| 225 | Int J Med Mushrooms. 2019;21(12):1151-1165. | NA | NA |
| 226 | Microrna. 2019;8(3):237-247 | NA | NA |
| 227 | Endocr Relat Cancer. 2019 Mar;26(3):321-337 | NA | NA |
| 228 | [J Bone Oncol. 2019 Jun; 16: 100232.](https://www-ncbi-nlm-nih-gov.proxy.insermbiblio.inist.fr/pmc/articles/PMC6434100/) | pet_ct_imaging | NA |
| 229 | J Immunol Res. 2019 May 9;2019:4260987 | x_ray_imaging | NA |
| 230 | Carcinogenesis. 2019 Apr 29;40(2):335-348 | caliper | other |
| 231 | *J Med Chem. 2019 Jun 13;62(11):5512-5521* | caliper | other |
| 232 | *Bioconjug Chem. 2019 Apr 17;30(4):1232-1243* | caliper | other |
| 233 | [BMC Cancer. 2019; 19: 645.](https://www-ncbi-nlm-nih-gov.proxy.insermbiblio.inist.fr/pmc/articles/PMC6604176/) | caliper | other |

**S1 Table: Summarize of the bibliographic search in the PubMed database from January 1^st^, 2019 to December 31^st^, 2019, using the followings terms: xenograft, breast cancer, tumor growth and mice.** * MR DWI = Diffusion-weighted magnetic resonance; PET CT = Positron emission tomography computing tomography; MD = missing data; NA = not applicable; unknown = information not accessible; Other = formulas did not describe a 3D representation
