# Supplementary material for: Tumor growth monitoring in breast cancer xenografts: A good technique for a strong ethic
Source: PLoS One. 2022 Sep 30;17(9):e0274886. doi: 10.1371/journal.pone.0274886 (PMC9524649; doi:10.1371/journal.pone.0274886)
Supplement: S2 Table — * MD = Missing data. (DOCX) [file pone.0274886.s003.docx]

| Protocol registration number | Mice identification | Day after engraftment | Length (mm) | Width (mm) | Height (mm) | Tumor mass (mg) | Length after exerisis (mm) | Width after exerisis (mm) | Height after exerisis (mm) |
| --- | --- | --- | --- | --- | --- | --- | --- | --- | --- |
| #2017031717108767 | C1_2 | 57 | 16.86 | 13.93 | 10 | 1900 | 20.62 | 15.85 | 9.7 |
| #2017031717108767 | C1_sm | 51 | 15.9 | 15 | 9.2 | 1900 | 17.59 | 16 | 7.81 |
| #2017031717108767 | C1_1g | 67 | 12.3 | 12.9 | 10 | MD | 16.49 | 16.8 | 11.7 |
| #2017031717108767 | C1 _5 | 57 | 16.6 | 14 | 8.38 | 2100 | 21 | 15.87 | 7.7 |
| #2017031717108767 | C2_1 | 47 | 17.53 | 14.76 | 9.3 | 2100 | 17.18 | 15.49 | 9.65 |
| #2017031717108767 | C2_2 | 60 | 16.69 | 13.49 | 5.88 | 2200 | 18.7 | 13.8 | 8.1 |
| #2017031717108767 | C2_3 | 57 | 15 | 13 | 10 | 1900 | 17.84 | 17.64 | 8.7 |
| #2017031717108767 | C2_ sm | 51 | 16.59 | 13.6 | 8.56 | 2300 | 20.59 | 16.38 | 2.3 |
| #2017031717108767 | C3_1g | 67 | 13.45 | 9.9 | 5.3 | MD | 12.9 | 11.11 | 3.8 |
| #2017031717108767 | C3_4 | 51 | 15 | 14.47 | 9.52 | 1700 | 14.3 | 15.5 | 7.8 |
| #2017031717108767 | C3_5 | 57 | 14.1 | 14.47 | 16.65 | MD | 14.98 | 16.88 | 10.55 |
| #2017031717108767 | C3_2d | 51 | 18.55 | 15.65 | 8.22 | 1900 | 19.82 | 16.6 | 8.29 |
| #2017031717108767 | C3_2g | 51 | 15.63 | 13.17 | 7.67 | 2300 | 18.85 | 17.37 | 10.48 |
| #2017031717108767 | C4_2 | 47 | 14.99 | 12.3 | 10.15 | 1700 | 17.06 | 17.15 | 9.4 |
| #2017031717108767 | C4_sm | 57 | 14.06 | 13.44 | 11.2 | 1600 | 16.94 | 16.97 | 11.3 |
| #2017031717108767 | C4_5 | 47 | 15.23 | 12.71 | 11.38 | 1900 | 17.68 | 17.5 | 9.45 |
| #2017031717108767 | C5_1 | 47 | 15.5 | 13.73 | 9.54 | 1700 | 17.55 | 15.48 | 8.89 |
| #2017031717108767 | C5_3 | 63 | 15.4 | 13.5 | 9.8 | 1700 | 17.14 | 15.1 | 8.3 |
| #2017031717108767 | C5_1d | 54 | 15.98 | 14.79 | 8.8 | 1800 | 19.43 | 14.46 | 8.5 |
| #2017031717108767 | C5_1g | 51 | 13.94 | 14.56 | 10.61 | 1600 | 14.55 | 14.73 | 9.26 |
| #2017031717108767 | C6_sm | 60 | 18.13 | 13.8 | 9.4 | MD | 21.4 | 15.36 | 8.5 |
| #2017031717108767 | C6_5 | 47 | 14.6 | 18.34 | 9.42 | 1800 | 14.85 | 18.55 | 12.58 |
| #2017031717108767 | C6_2d | 54 | 19.23 | 14.37 | 8.06 | 2100 | 19.5 | 15.31 | 8.58 |
| #2017031717108767 | C6_2g | 47 | 15.6 | 13.8 | 10.2 | 1900 | 17.7 | 18.3 | 10.63 |
| #2017031717108767 | C7_2 | 51 | 16.48 | 14.58 | 8.89 | 1600 | 16.43 | 18.43 | 8.9 |
| #2017031717108767 | C7_1g | 51 | 14.56 | 14.6 | 9.76 | 1700 | 15.67 | 17.42 | 11.9 |
| #2017031717108767 | C7_sm | 47 | 15.34 | 14.9 | 10.9 | 2200 | 16.31 | 17.46 | 10.68 |
| #2017031717108767 | C7_4 | 51 | 14.9 | 16.64 | 11 | 2100 | 17.63 | 19.75 | 10.79 |
| #2017031717108767 | C8_5 | 47 | 12.9 | 12.7 | 9.5 | 1900 | 18.7 | 17.7 | 9.58 |
| #2017031717108767 | C8_2d | 47 | 16.09 | 15.27 | 10 | 2500 | 18 | 19.7 | 9.47 |
| #2017031717108767 | C8_2g | 51 | 13.4 | 11 | 8.7 | 1600 | 18 | 13.54 | 8.23 |
| #20181213155790720 | C1_B | 28 | 10.65 | 9.79 | 4.93 | 360 | MD | MD | MD |
| #20181213155790720 | C1_1 | 28 | 11.1 | 10.67 | 6.87 | 514 | MD | MD | MD |
| #20181213155790720 | C1_1b | 21 | 8.95 | 4.5 | 4.67 | MD | MD | MD | MD |
| #20181213155790720 | C1_1b | 25 | 9.72 | 5.42 | 6.3 | MD | MD | MD | MD |
| #20181213155790720 | C1_1b | 28 | 9.42 | 6.85 | 5.77 | MD | MD | MD | MD |
| #20181213155790720 | C1_1b | 32 | 11.82 | 7.31 | 6.31 | MD | MD | MD | MD |
| #20181213155790720 | C1_1b | 35 | 10.31 | 7.86 | 7.71 | MD | MD | MD | MD |
| #20181213155790720 | C1_1b | 39 | 10.14 | 12.13 | 9.68 | MD | MD | MD | MD |
| #20181213155790720 | C1_1b | 42 | 10.53 | 12.76 | 8.74 | MD | MD | MD | MD |
| #20181213155790720 | C1_1b | 46 | 16.04 | 13.59 | 9.6 | MD | MD | MD | MD |
| #20181213155790720 | C1_1d | 21 | 13.05 | 9.22 | 5.87 | MD | MD | MD | MD |
| #20181213155790720 | C1_1d | 25 | 14.45 | 9.59 | 6.57 | MD | MD | MD | MD |
| #20181213155790720 | C1_1d | 32 | 12.8 | 8.81 | 8.78 | MD | MD | MD | MD |
| #20181213155790720 | C1_1d | 35 | 12.77 | 11 | 8.7 | MD | MD | MD | MD |
| #20181213155790720 | C1_1d | 39 | 14.07 | 10.82 | 11.17 | MD | MD | MD | MD |
| #20181213155790720 | C1_N | 28 | 11.53 | 8.21 | 6.73 | 366 | MD | MD | MD |
| #20181213155790720 | C4_1 | 28 | 10.69 | 11.21 | 9.3 | 443 | MD | MD | MD |
| #20181213155790720 | C4_3 | 28 | 12.71 | 9.83 | 7.73 | 495 | MD | MD | MD |
| #20181213155790720 | C4_N | 28 | 10.11 | 10.13 | 7.91 | 390 | MD | MD | MD |
| #20181213155790720 | C3_3 | 29 | 12.93 | 11.83 | 7.27 | 764 | MD | MD | MD |
| #20181213155790720 | C10_2 | 29 | 9.14 | 9.99 | 6.26 | 387 | MD | MD | MD |
| #20181213155790720 | C7_N | 29 | 13.17 | 10.47 | 8.95 | 553 | MD | MD | MD |
| #20181213155790720 | C8_B | 29 | 10.19 | 12.68 | 7.55 | 429 | MD | MD | MD |
| #20181213155790720 | C8_1 | 29 | 10.9 | 11.67 | 6.24 | 514 | MD | MD | MD |
| #20181213155790720 | C5_N | 35 | 13.27 | 8.83 | 7.77 | 645 | MD | MD | MD |
| #20181213155790720 | C5_2 | 35 | 15.29 | 9.65 | 6.92 | 872 | MD | MD | MD |
| #20181213155790720 | C9_3 | 34 | 9.57 | 8.65 | 5.79 | 435 | MD | MD | MD |
| #20181213155790720 | C9_N | 34 | 8.96 | 8.96 | 6.29 | 457 | MD | MD | MD |
| #20181213155790720 | C1_3 | 33 | 11.15 | 7.22 | 6.86 | 371 | MD | MD | MD |
| #20181213155790720 | C3_2 | 33 | 8.89 | 8.28 | 5.07 | 304 | MD | MD | MD |
| #20181213155790720 | C3_1 | 33 | 7.08 | 6.09 | 5.38 | 155 | MD | MD | MD |
| #20181213155790720 | C3_B | 33 | 8.4 | 7.09 | 4.5 | 194 | MD | MD | MD |
| #20181213155790720 | C3_N | 33 | 9.45 | 8.46 | 5.46 | 425 | MD | MD | MD |
| #20181213155790720 | C6_B | 33 | 9.47 | 10.33 | 7.92 | 485 | MD | MD | MD |
| #20181213155790720 | C7_B | 33 | 10.17 | 9.45 | 7.69 | 549 | MD | MD | MD |
| #20181213155790720 | C7_1 | 33 | 8.27 | 6.22 | 4.88 | 208 | MD | MD | MD |
| #20181213155790720 | C7_2 | 33 | 10.78 | 8.95 | 7.32 | 541 | MD | MD | MD |
| #20181213155790720 | C4_B | 33 | 8.7 | 10.02 | 6.21 | 266 | MD | MD | MD |
| #20181213155790720 | C4_2 | 33 | 11.87 | 11.22 | 8.15 | 577 | MD | MD | MD |
| #20181213155790720 | C5_3 | 33 | 15 | 8.12 | 6.69 | 375 | MD | MD | MD |
| #20181213155790720 | C5_B | 34 | 11.43 | 9.27 | 6.51 | 549 | MD | MD | MD |
| #20181213155790720 | C5_1 | 34 | 11.81 | 9.87 | 5.75 | 449 | MD | MD | MD |
| #20181213155790720 | C1_2 | 34 | 9.45 | 9.85 | 5.76 | 563 | MD | MD | MD |
| #20181213155790720 | C1_2b | 39 | 13.56 | 12.84 | 8.1 | 1440 | MD | MD | MD |
| #20181213155790720 | C1_2b | 21 | 7.6 | 8.3 | 6 | MD | MD | MD | MD |
| #20181213155790720 | C1_2b | 25 | 9.43 | 9.2 | 7.2 | MD | MD | MD | MD |
| #20181213155790720 | C1_2b | 28 | 10.9 | 10.4 | 7.58 | MD | MD | MD | MD |
| #20181213155790720 | C1_2b | 32 | 10.23 | 9.77 | 8.15 | MD | MD | MD | MD |
| #20181213155790720 | C1_2b | 35 | 11.5 | 12.49 | 9.27 | MD | MD | MD | MD |
| #20181213155790720 | C1_3 | 32 | 17.24 | 13.16 | 10.58 | 2831 | MD | MD | MD |
| #20181213155790720 | C1_3 | 21 | 10.97 | 8.47 | 6.67 | MD | MD | MD | MD |
| #20181213155790720 | C1_3 | 25 | 12.53 | 14.37 | 10.17 | MD | MD | MD | MD |
| #20181213155790720 | C1_3 | 28 | 15.29 | 13.17 | 8.6 | MD | MD | MD | MD |
| #20181213155790720 | C1_4 | 35 | 9.25 | 9.02 | 7.85 | 849 | MD | MD | MD |
| #20181213155790720 | C1_4 | 21 | 7.66 | 8.5 | 6.56 | MD | MD | MD | MD |
| #20181213155790720 | C1_4 | 25 | 9.57 | 7.94 | 7.72 | MD | MD | MD | MD |
| #20181213155790720 | C1_4 | 28 | 9.7 | 9 | 6.97 | MD | MD | MD | MD |
| #20181213155790720 | C1_4 | 32 | 8.72 | 8.68 | 7.46 | MD | MD | MD | MD |
| #20181213155790720 | C1_SM | 21 | 5.65 | 6.43 | 4.78 | MD | MD | MD | MD |
| #20181213155790720 | C1_SM | 25 | 5.7 | 6.47 | 4.63 | MD | MD | MD | MD |
| #20181213155790720 | C1_SM | 28 | 7.06 | 7.01 | 5.5 | MD | MD | MD | MD |
| #20181213155790720 | C1_SM | 32 | 7.28 | 7.49 | 6.24 | MD | MD | MD | MD |
| #20181213155790720 | C1_SM | 35 | 8.06 | 9.51 | 5.97 | MD | MD | MD | MD |
| #20181213155790720 | C1_SM | 39 | 10.27 | 9.07 | 6.3 | MD | MD | MD | MD |
| #20181213155790720 | C1_SM | 42 | 9.09 | 10.68 | 6.06 | MD | MD | MD | MD |
| #20181213155790720 | C1_SM | 46 | 14.05 | 12.15 | 8.58 | MD | MD | MD | MD |
| #20181213155790720 | C2_1 | 21 | 3.92 | 3.92 | 3.52 | MD | MD | MD | MD |
| #20181213155790720 | C2_1 | 25 | 4.18 | 4.65 | 3.52 | MD | MD | MD | MD |
| #20181213155790720 | C2_1 | 28 | 3.96 | 4.96 | 3.74 | MD | MD | MD | MD |
| #20181213155790720 | C2_1 | 32 | 4.88 | 5.6 | 4.17 | MD | MD | MD | MD |
| #20181213155790720 | C2_1 | 35 | 5.59 | 5.87 | 4.64 | MD | MD | MD | MD |
| #20181213155790720 | C2_1 | 39 | 6.65 | 6.21 | 4.66 | MD | MD | MD | MD |
| #20181213155790720 | C2_1 | 42 | 6.6 | 7.12 | 4.82 | MD | MD | MD | MD |
| #20181213155790720 | C2_1 | 46 | 8.57 | 8.57 | 5.88 | MD | MD | MD | MD |
| #20181213155790720 | C2_1 | 49 | 9.4 | 8.03 | 5.36 | MD | MD | MD | MD |
| #20181213155790720 | C2_1 | 53 | 7.97 | 8.32 | 5.89 | MD | MD | MD | MD |
| #20181213155790720 | C2_1 | 56 | 8.64 | 8.11 | 5.84 | MD | MD | MD | MD |
| #20181213155790720 | C2_1 | 60 | 8.21 | 9.93 | 4.26 | MD | MD | MD | MD |
| #20181213155790720 | C2_1 | 63 | 9.88 | 8.7 | 6.12 | MD | MD | MD | MD |
| #20181213155790720 | C2_1 | 67 | 10.2 | 9.05 | 5.68 | MD | MD | MD | MD |
| #20181213155790720 | C2_1 | 70 | 10.2 | 9.52 | 6.47 | MD | MD | MD | MD |
| #20181213155790720 | C2_1D | 60 | 11.19 | 11.44 | 6.35 | MD | MD | MD | MD |
| #20181213155790720 | C2_1D | 56 | 10.13 | 10.37 | 4.93 | MD | MD | MD | MD |
| #20181213155790720 | C2_1D | 53 | 10.37 | 10.47 | 5.89 | MD | MD | MD | MD |
| #20181213155790720 | C2_1D | 49 | 10.94 | 11.26 | 5.76 | MD | MD | MD | MD |
| #20181213155790720 | C2_1D | 46 | 10.94 | 11.4 | 5.81 | MD | MD | MD | MD |
| #20181213155790720 | C2_1D | 42 | 7.51 | 8.76 | 5.14 | MD | MD | MD | MD |
| #20181213155790720 | C2_1D | 39 | 7.46 | 8.8 | 5.74 | MD | MD | MD | MD |
| #20181213155790720 | C2_1D | 35 | 6.38 | 7.88 | 5.28 | MD | MD | MD | MD |
| #20181213155790720 | C2_1D | 32 | 5.21 | 7.47 | 4.17 | MD | MD | MD | MD |
| #20181213155790720 | C2_1D | 28 | 5.46 | 6.95 | 4.18 | MD | MD | MD | MD |
| #20181213155790720 | C2_1D | 25 | 4.65 | 6.54 | 3.51 | MD | MD | MD | MD |
| #20181213155790720 | C2_1D | 21 | 4.25 | 5.8 | 4.26 | MD | MD | MD | MD |
| #20181213155790720 | C2_SM | 21 | 5.24 | 5.59 | 5.7 | MD | MD | MD | MD |
| #20181213155790720 | C2_SM | 25 | 6.57 | 6.5 | 4.53 | MD | MD | MD | MD |
| #20181213155790720 | C2_SM | 28 | 6.75 | 6.87 | 5.7 | MD | MD | MD | MD |
| #20181213155790720 | C2_SM | 32 | 6.77 | 7.65 | 6.06 | MD | MD | MD | MD |
| #20181213155790720 | C2_SM | 35 | 7.78 | 8.46 | 6.28 | MD | MD | MD | MD |
| #20181213155790720 | C2_SM | 39 | 8.83 | 9.61 | 7.75 | MD | MD | MD | MD |
| #20181213155790720 | C2_SM | 42 | 9.4 | 10.03 | 6.51 | MD | MD | MD | MD |
| #20181213155790720 | C2_SM | 46 | 13.22 | 11.95 | 9.29 | MD | MD | MD | MD |
| #20181213155790720 | C2_SM | 49 | 11.4 | 12.82 | 9 | MD | MD | MD | MD |
| #20181213155790720 | C2_SM | 53 | 12.38 | 14.1 | 7.55 | MD | MD | MD | MD |
| #20181213155790720 | C2_1G | 32 | 14.68 | 9.27 | 7.85 | 984 | MD | MD | MD |
| #20181213155790720 | C2_1G | 21 | 10.75 | 8.02 | 6.17 | MD | MD | MD | MD |
| #20181213155790720 | C2_1G | 25 | 11.5 | 9.09 | 6.6 | MD | MD | MD | MD |
| #20181213155790720 | C2_1G | 28 | 12.3 | 9.86 | 4.2 | MD | MD | MD | MD |
| #20181213155790720 | C2_2G | 32 | 11 | 7.74 | 6.49 | 1011 | MD | MD | MD |
| #20181213155790720 | C2_2G | 28 | 10.06 | 9.38 | 6.17 | MD | MD | MD | MD |
| #20181213155790720 | C2_2G | 25 | 9.35 | 9.26 | 5.64 | MD | MD | MD | MD |
| #20181213155790720 | C2_2G | 21 | 7.4 | 9.4 | 6.69 | MD | MD | MD | MD |
| #20181213155790720 | C2_2D | 39 | 13.21 | 13.77 | 11.68 | 2759 | MD | MD | MD |
| #20181213155790720 | C2_2D | 35 | 10.95 | 12.88 | 9.33 | MD | MD | MD | MD |
| #20181213155790720 | C2_2D | 32 | 10.82 | 11.93 | 10.78 | MD | MD | MD | MD |
| #20181213155790720 | C2_2D | 28 | 10.84 | 10.41 | 8.3 | MD | MD | MD | MD |
| #20181213155790720 | C2_2D | 25 | 10.39 | 9.59 | 7.95 | MD | MD | MD | MD |
| #20181213155790720 | C2_2D | 21 | 9.22 | 10.05 | 6.93 | MD | MD | MD | MD |
| #20181213155790720 | C3_1 | 39 | 12.53 | 12.95 | 7.89 | 1304 | MD | MD | MD |
| #20181213155790720 | C3_3 | 39 | 13.86 | 12.15 | 10.42 | 1765 | MD | MD | MD |
| #20181213155790720 | C3_DG | 39 | 15.44 | 12.37 | 9.32 | 2394 | MD | MD | MD |
| #20181213155790720 | C4_SM | 28 | 6.75 | 8.54 | 5.19 | 205 | MD | MD | MD |
| #20181213155790720 | C4_2G | 28 | 15.36 | 9.83 | 7.57 | 2130 | MD | MD | MD |
| #20181213155790720 | C4_DG | 32 | 10.87 | 9.71 | 6.77 | 847 | MD | MD | MD |
| #20181213155790720 | C5_SM | 39 | 12.74 | 11.11 | 7.84 | 1200 | MD | MD | MD |
| #20181213155790720 | C5_1D | 28 | 6.44 | 9.36 | 5.9 | 459 | MD | MD | MD |
| #20181213155790720 | C5_2D | 39 | 11.9 | 12.6 | 13.75 | 2627 | MD | MD | MD |
| #20181213155790720 | C5_2G | 39 | 12.8 | 11.63 | 9.18 | 2256 | MD | MD | MD |
| #20181213155790720 | C6_SM | 39 | 13.58 | 14.7 | 10.07 | 2976 | MD | MD | MD |
| #20181213155790720 | C6_1 | 35 | 11.48 | 10.92 | 7.37 | 1305 | MD | MD | MD |
| #20181213155790720 | C6_2 | 39 | 11.88 | 10.74 | 7.15 | 889 | MD | MD | MD |
| #20181213155790720 | C6_DG | 35 | 14.63 | 15.24 | 11.66 | 2453 | MD | MD | MD |
| #20181213155790720 | C7_SM | 39 | 11.11 | 13.2 | 6.8 | 1617 | MD | MD | MD |
| #20181213155790720 | C7_1 | 32 | 8.95 | 8.36 | 6.37 | 543 | MD | MD | MD |
| #20181213155790720 | C7_DG | 28 | 8 | 11.09 | 7.7 | 518 | MD | MD | MD |
| #20181213155790720 | C7_2D | 28 | 10.91 | 9.86 | 8.21 | 756 | MD | MD | MD |
| #20181213155790720 | C8_1 | 28 | 9.1 | 10.8 | 6.95 | 646 | MD | MD | MD |
| #20181213155790720 | C8_1G | 39 | 15.64 | 13.75 | 11.19 | 2789 | MD | MD | MD |

**S2 Table: Summarize of relevant animal data from protocol registration #2017031717108767 and #20181213155790720** (n=87). * MD = Missing data
