# Supplementary material for: Tumor growth monitoring in breast cancer xenografts: A good technique for a strong ethic
Source: PLoS One. 2022 Sep 30;17(9):e0274886. doi: 10.1371/journal.pone.0274886 (PMC9524649; doi:10.1371/journal.pone.0274886)
Supplement: S3 Table — α_0 is the specific growth rate (in 1/days), β is the parameter driving the exponential decrease of the proliferation rate (in 1/days), and σ is the error model parameter. R.S.E. is the relative standard error on parameter estimation. CV is the coefficient of variation expressed as 100∙exp(ω^2 − 1). (DOCX) [file pone.0274886.s004.docx]

| Formula | Parameter | Estimate | R.S.E. (%) | CV (%) |
| --- | --- | --- | --- | --- |
| 1 | $\alpha_{0}$ | 0.199 | 6.3 | 11.9 |
|  | $\beta$ | 0.016 | 13.3 | 34.9 |
|  | $\sigma$ | 0.193 | 8.9 | - |
| 2 | $\alpha_{0}$ | 0.235 | 3.1 | 7.4 |
|  | $\beta$ | 0.019 | 8.2 | 24.1 |
|  | $\sigma$ | 0.193 | 8.8 | - |
| 3 | $\alpha_{0}$ | 0.207 | 10.0 | 22.9 |
|  | $\beta$ | 0.015 | 21.0 | 56.5 |
|  | $\sigma$ | 0.194 | 9.2 | - |
| 4 | $\alpha_{0}$ | 0.266 | 2.7 | 6.7 |
|  | $\beta$ | 0.023 | 6.9 | 20.0 |
|  | $\sigma$ | 0.218 | 9.0 | - |
| 5 | $\alpha_{0}$ | 0.247 | 2.9 | 8.6 |
|  | $\beta$ | 0.020 | 8.9 | 27.8 |
|  | $\sigma$ | 0.208 | 8.7 | - |
| 6 | $\alpha_{0}$ | 0.240 | 11.3 | 23.7 |
|  | $\beta$ | 0.016 | 21.2 | 46.8 |
|  | $\sigma$ | 0.216 | 8.7 | - |
| 7 | $\alpha_{0}$ | 0.220 | 10.2 | 21.1 |
|  | $\beta$ | 0.015 | 20.1 | 46.0 |
|  | $\sigma$ | 0.218 | 8.7 | - |

**S3 Table: Parameter estimates of the Gompertz model obtained fitting the data obtained with different formulations.** $\boldsymbol{\alpha}_{\boldsymbol{0}}$ **is the specific growth rate (in 1/days),** $\boldsymbol{\beta}$ **is the parameter driving the exponential decrease of the proliferation rate (in 1/days), and** $\boldsymbol{\sigma}$ **is the error model parameter. R.S.E. is the relative standard error on parameter estimation. CV is the coefficient of variation expressed as** $\boldsymbol{100\cdot exp(}\boldsymbol{\omega}^{\boldsymbol{2}}\boldsymbol{-1)}$**.**
