# Supplementary material for: Tumor growth monitoring in breast cancer xenografts: A good technique for a strong ethic
Source: PLoS One. 2022 Sep 30;17(9):e0274886. doi: 10.1371/journal.pone.0274886 (PMC9524649; doi:10.1371/journal.pone.0274886)
Supplement: S4 Table — *MRE = mean relative error, MAE = mean absolute error, r2 = correlation coefficient of regression line, a = slope of regression line. (DOCX) [file pone.0274886.s005.docx]

| Formula | MRE | MAE | R2 | a | b | Error Model Parameter |
| --- | --- | --- | --- | --- | --- | --- |
| Formula 1 | 0.391 | 579.65 | 0.765 | 1.85439 | -23.944 | 0.193 |
| Formula 2 | 0.335 | 313.79 | 0.765 | 0.97096 | -23.9937 | 0.193 |
| Formula 3 | 0.272 | 343.92 | 0.762 | 1.07831 | 124.638 | 0.194 |
| Formula 4 | 0.282 | 368.33 | 0.659 | 1.11282 | 146.6198 | 0.218 |
| Formula 5 | 0.275 | 368.78 | 0.678 | 1.15742 | 131.25 | 0.208 |
| Formula 6 | 3.07 | 3253.54 | 0.659 | 0.24729 | 146.6198 | 0.216 |
| Formula 7 | 0.761 | 718.14 | 0.659 | 0.58267 | 146.6198 | 0.218 |

**S4 Table: Volume estimation data (i.e., MRE, MAE, r2, *a*, *b* and Error Model Parameter) for Formula 1 (F1), Formula 2 (F2) Formula 3 (F3), Formula 4 (F4), Formula 5 (F5), Formula 6 (F6) and Formula 7 (F7).** *MRE = mean relative error, MAE = mean absolute error, r2 = correlation coefficient of regression line, *a* = slope of regression line, *b* = intercept of regression line.
